# Genome variation of Sporothrix schenckii and Sporothrix brasiliensis

**DOI:** 10.1371/journal.pntd.0014203

**Published:** 2026-09-21

**Authors:** Ujwal R. Bagal, Amanda R. Santos, Rodrigo Almeida Paes, Lucas Gomes de Brito Alves, Luz Rocio Chamorro, Lindsay A. Parnell, Jose Pereira Brunelli, Nancy A. Chow, Jan Pohl, Vanessa Rabello Brito, Vanice Rodrigues Poester, Bram Spruijtenburg, Larissa Fernandes, Bridget M. Barker, Jason E. Stajich, Maria Sueli Felipe, Johanna Rhodes, Melissa Orzechowski Xavier, Daniel R. Matute, Rosely Zancope, Marcus de Melo Teixeira

**Affiliations:** 1 Mycotic Diseases Branch, Centers for Disease Control and Prevention, Atlanta, Georgia, United States of America; 2 Biotechnology Core Facility Branch, Centers for Disease Control and Prevention, Atlanta, Georgia, United States of America; 3 Instituto Nacional de Infectologia Evandro Chagas: Rio de Janeiro, Rio de Janeiro, Brazil; 4 Núcleo de Medicina Tropical, Faculty of Medicine, University of Brasília, Brasília, Distrito Federal, Brazil; 5 Genomic Sciences and Biotechnology Graduate Program, Universidade Católica de Brasília (UCB), Brasília, Distrito Federal, Brazil; 6 Ministry of Public Health and Social Welfare, Asuncion, Paraguay; 7 School of Medicine, Federal University of Rio Grande (FURG), Rio Grande, Rio Grande do Sul, Brazil; 8 Radboudumc-CWZ Center of Expertise for Mycology, Nijmegen, The Netherlands; 9 Department of Medical Microbiology and Immunology, Canisius-Wilhelmina Hospital (CWZ)/Dicoon, Nijmegen, The Netherlands; 10 Faculty of Ceilandia, University of Brasilia, Brasilia, Distrito Federal, Brazil; 11 Pathogen and Microbiome Institute, Northern Arizona University, Flagstaff, Arizona, United States of America; 12 Department of Microbiology and Plant Pathology, University of California-Riverside, Riverside, California, United States of America; 13 School of Biosciences, University of Birmingham, Birmingham, United Kingdom; 14 University of North Carolina, Chapel Hill, North Carolina, United States of America; FIOCRUZ: Fundacao Oswaldo Cruz, BRAZIL

## Abstract

Sporotrichosis, a subcutaneous mycosis caused by dimorphic fungi of the *Sporothrix* genus, has become a major zoonotic epidemic in South America, primarily driven by *Sporothrix brasiliensis*. To elucidate the genomic basis of its emergence and antifungal adaptation, we analyzed whole-genome sequences from 95 *Sporothrix* isolates, integrating single-nucleotide polymorphism (SNP), copy number variation (CNV), and genome-wide association (GWAS) analyses. Comparative genomics revealed 610,242 SNPs within *S. brasiliensis* and 1,474,627 within *S. schenckii*, confirming a marked disparity in intraspecific diversity. Phylogenomic tree inference resolved six well-supported *S. brasiliensis* clades with limited internal divergence, reflecting recent population expansion, while *S. schenckii* displayed deep phylogeographic structure separating North and South American lineages. CNV profiling identified 158 affected genes in *S. brasiliensis* (60 gains, 98 losses) and 88 in *S. schenckii* (54 gains, 34 losses), concentrated near sub-telomeric regions. In *S. brasiliensis*, gains were enriched for kinases and intracellular trafficking functions, whereas losses involved genes related to translation and primary metabolism, suggesting regulatory reinforcement coupled with metabolic streamlining. A GWAS of itraconazole resistance identified 81 SNPs distributed across multiple scaffolds, with many located within genes related with transport, signaling, and redox balance, supporting a polygenic basis for azole response. The implication of these alleles with itraconazole resistance warrants experimental followup. Together, these results highlight distinct evolutionary strategies of closely related *Sporothrix* species and pinpoint genomic changes potentially associated with the emergence and drug tolerance of *S. brasiliensis*.

## Author summary

Sporotrichosis is a fungal disease that affects humans and animals and has become an important public health problem in South America. The disease is often transmitted from infected cats to humans and is mainly caused by *Sporothrix brasiliensis*, although related species such as *Sporothrix schenckii* also cause human infections. In this study, we analyzed whole-genome data from *S. brasiliensis* and *S. schenckii* isolates to better understand how these fungi are evolving, spreading, and responding to antifungal treatment. We found that *S. brasiliensis* has a more clonal population structure, consistent with recent epidemic expansion, whereas *S. schenckii* shows deeper geographic separation between North and South American lineages. We also identified genome regions affected by copy number variation, suggesting that structural changes may contribute to adaptation in these fungi. Finally, we detected genetic variants associated with itraconazole response. The contribution of these alleles will need to be followed up with functional genetics assays. These findings provide a genomic framework for surveillance of sporotrichosis and for future studies on fungal emergence and antifungal resistance.

## Introduction

Fungal diseases are increasingly recognized as a major threat to human and animal health, with an estimated one billion individuals affected worldwide each year [1]. Among these, sporotrichosis has emerged as a critical and expanding public health concern, particularly in South America, where zoonotic transmission from domestic animals, especially cats, has fueled sustained outbreaks of unprecedented scale [2]. Sporotrichosis is a subcutaneous mycosis caused by thermally dimorphic fungi of the genus *Sporothrix*, with most human infections attributed to members of the pathogenic clade, a monophyletic lineage comprising *Sporothrix schenckii* sensu stricto, *Sporothrix brasiliensis*, *Sporothrix globosa*, and *Sporothrix luriei* [3]. These fungi inhabit soil, decaying plant material, and organic debris, with transmission primarily occurring through traumatic implantation into the skin [4]. Historically, sporotrichosis was considered mainly a sapronotic, occupation-associated infection acquired through traumatic inoculation of *Sporothrix* spp. from soil and plant material [5]. However, *Sporothrix brasiliensis* has emerged as a highly virulent zoonotic pathogen, driving large-scale epidemics of cat-associated sporotrichosis across Brazil, Argentina, Paraguay, Chile, and Uruguay, and has become the dominant etiological agent in South America [6]. In parallel, zoonotic transmission by *Sporothrix schenckii* has emerged in Southeast Asia and the United States, reflecting a convergent shift toward feline-associated transmission [7,8].

The clinical manifestations of sporotrichosis vary depending on host susceptibility and fungal species. In humans, the disease typically presents as a localized cutaneous or lymphocutaneous infection, but in immunocompromised individuals, it can progress to disseminated or extracutaneous forms, with pulmonary and osteoarticular involvement [9]. In contrast, feline infections caused by *S. brasiliensis* are often severe, with high fungal burdens, extensive ulcerative lesions, and frequent systemic dissemination. Zoonotic transmission from infected cats to humans has been a major driver of the sporotrichosis epidemic in Brazil, where *S. brasiliensis* has been implicated in nearly all cases [4,10,11]. Unlike other *Sporothrix* species, *S. brasiliensis* isolates show enhanced virulence, higher transmissibility, and increased antifungal resistance [12–15].

Recent genomic studies have provided insights into the evolutionary relationships among *Sporothrix* species, demonstrating that *S. brasiliensis* and *S. schenckii* are sister species, and diverged between 3.8 and 4.9 million years ago [12]. The two species differ by approximately 442,000 single-nucleotide polymorphisms (SNPs), and the dyad’s most closely related species is *S. globosa* [12,16,17]. Within *S. brasiliensis*, population genomic analyses have identified several geographically restricted lineages in Brazil, suggesting diverse population structure [4,11,18-20]. Despite these findings, the underlying factors driving the epidemic spread of *S. brasiliensis* remain poorly understood. Specifically, little is known about how genetic variation is partitioned across the geographic range of *S. brasiliensis*. Additionally, although *S. brasiliensis* exhibits phenotypic variation such as pigmentation and growth rate, the emergence of antifungal resistance represents one of the most concerning adaptive trajectories in this pathogen [2,21–25]. Yet, the molecular mechanisms underlying this trait remain largely unexplored (but see [13,26]).

In this study, we applied whole-genome sequencing (WGS) to investigate intraspecific genetic variation, population structure, and large-scale structural variation, including copy number variations (CNVs), in clinical isolates of *Sporothrix schenckii* and *S. brasiliensis*. By integrating antifungal susceptibility testing with comparative genomic and CNV analyses, we aimed to identify genomic features associated with antifungal resistance, pathogenicity, and host adaptation. Our structural analyses reveal species and lineage-specific CNV landscapes, marked by contrasting patterns of gene gains and losses, heterogeneous chromosomal blocks of amplification and deletion, and distinct population-level CNV signatures. These genomic architectures highlight fundamentally different modes of genome plasticity between *S. brasiliensis* and *S. schenckii*. Given the increasing burden of sporotrichosis, particularly in South America, our findings provide critical insights into the genomic mechanisms underlying the emergence and diversification of *S. brasiliensis*, with direct implications for public health surveillance, antifungal treatment strategies, and the evolutionary dynamics of pathogenicity.

## Methods

### Genome sequencing

We sequenced 13 new *Sporothrix* isolates (11 *S. brasiliensis* and 2 *S. schenckii*), ten strains of which were isolated from Brazil and three from Paraguay. All isolates were cultured in Brain Heart Infusion (BHI) broth (Becton Dickinson and Company, MD, USA) at 37°C for seven days. Following incubation, genomic DNA was extracted from the yeast-phase cells using a previously established protocol [27]. DNA integrity was assessed by electrophoresis on 1% agarose gel with a DNA mass ladder, and by spectrophotometric analysis using a NanoDrop 2000 (Thermo Scientific).

We used these DNA extractions to sequence the genome of each isolate. Sequencing libraries were prepared from 1 µg of high-quality genomic DNA per sample using the KAPA HyperPrep Kit (Roche, Basel, Switzerland), following the manufacturers protocol optimized for Illumina sequencing platforms. Each library was uniquely indexed with 8 bp nucleotide barcodes, and final concentrations were quantified using the KAPA Library Quantification Kit (Kapa Biosystems) on a 7900HT real-time PCR system (Life Technologies). Libraries were then paired-end sequenced with a read length of 250 bp, using v4 chemistry on the Illumina NovaSeq 6000 system (Illumina, San Diego, CA).

### Genomic data

We also incorporated previously published genomic data from *S. brasiliensis* and *S. schenckii* [12,16,17]. S1 Table lists all the isolates used in the study, along with their geographic origin and accession numbers. In total, we included 82 previously sequenced *Sporothrix* genomes - 73 *S. brasiliensis* isolates and 9 *S. schenckii* isolates*.* The *S. brasiliensis* samples included both human (69%) and animal (30%) cases of sporotrichosis. All *S. schenckii* isolates were retrieved from human samples. The isolates were collected over a span of 24 years (from 1998 to 2022), although ten samples (9 *S. brasiliensis* and 1 *S. schenckii*) lacked specific collection date information. Of these, 73 *S. brasiliensis* isolates were from Brazil and their genomic sequences were retrieved from the NCBI SRA database (S1 Table). The 9 previously sequenced *S. schenckii* isolates originated from Brazil, Colombia and the USA.

### Data preprocessing, read mapping, and variant calling

All FASTQ files were processed using the North Arizona SNP pipeline (NASP v1.0) [28]. NASP uses trimmomatic (v0.35) [29] for raw reads trimming, Samtools (v1.2) [30] for reference genome indexing, NUCmer aligner from MUMmer (v4) [31,32] to identify duplicate regions and BWA-MEM (v0.7.7) [33] for mapping reads to the reference genome. For the whole-genome SNP phylogeny, reads from all *Sporothrix* isolates were aligned to the *S. brasiliensis* reference genome GCF_000820605.1, allowing SNPs from both *S. brasiliensis* and *S. schenckii* to be called within a single reference-coordinate system. For population genetic analyses, however, SNPs were called separately within each species to minimize reference-mapping bias: *S. brasiliensis* reads were mapped to GCF_000820605.1, whereas *S. schenckii* reads were mapped to the *S. schenckii* reference genome GCA_000961545.1. The resulting species-specific BAM files and SNP datasets were used independently for Copy Number Variation, PCA and fastSTRUCTURE analyzes. Sequencing reads from each species were aligned against their respective reference genomes, *S. brasiliensis* (GCF_000820605.1) and *S. schenckii* (GCA_000961545.1). The final dataset comprised a total of 95 isolates. Next, we used the resulting BAM files to call variant sites using GATK (v3.6) [34]. We first applied the realignerTargetCreator and IndelRealigner tool from GATK to identify and generate a target interval list for local realignment. The resulting realigned BAM files were then used as input for the UnifiedGenotyper, specifying haploid ploidy and an estimated heterozygosity value of 0.01. Following variant calling, SNPs were hard-filtered using the GATK VariantFiltration tool. Retained SNPs were required to meet the following criteria: Quality by Depth (QD) ≥ 2.0, Fisher Strand Bias (FS) ≤ 60.0, Mapping Quality (MQ) ≥ 30.0, MQRankSum ≥ −12.5, and ReadPosRankSum ≥ −8.0. We further excluded SNPs with read depth <10 × , variant allele frequency <90% and SNPs located within putative duplicated regions identified by NUCmer were discarded. The mating-type idiomorphs of both *Sporothrix* genomes were predicted by mapping sequencing reads against the full-length nucleotide sequences of the MAT1–1 and MAT1–2 loci [11] using the Minimap2 tool [35] and default parameters.

### Phylogenomics and population genetics

Maximum likelihood (ML) phylogenetic trees were constructed using IQ-TREE2 [36], to investigate the genealogical relationships among the *Sporothrix* isolates. Within IQ-TREE2, the ModelFinder [37] feature was used to determine the optimal nucleotide substitution model by employing the TEST function to systematically evaluate and select the best-fit evolutionary model based on the Bayesian Information Criterion (BIC). Phylogenies were inferred from whole-genome single nucleotide polymorphisms (SNPs) as well as from individual scaffolds derived from *S. brasiliensis* reference genomes to evaluate genealogical concordance among genome segments. Branch support was estimated using three complementary approaches: ultrafast bootstrap approximation (UFBoot) [38] and approximate likelihood-ratio tests (aLRT) [39], each performed using 1,000 replicates and the Bayesian-like transformation of aLRT (aBayes) metric to provide reliable confidence estimates for tree topology [40]. All resulting trees were visualized using FigTree v1.4.2 (http://tree.bio.ed.ac.uk/software/figtree/).

The population structure was evaluated using two complementary population genetic approaches: principal component analysis (PCA) and model-based clustering. To further assess clustering, fastStructure v1.0 [41] and ADMIXTURE v1.3.0 [42], were used to estimate the most likely number of genetic clusters represented in the isolate dataset. For these analyses, genomic data were formatted using Plink1 [43] to generate the required input files, including.bed (binary genotype data with individual sample identifiers),.bim (SNP marker positions and annotations),.fam (sample metadata),.map (genomic positions of SNPs), and.ped (pedigree and genotype data). The number of populations (K) was evaluated across a range from 1 to 12, and the ChooseK.py script from fastStructure was used to determine the most appropriate number of clusters. Phylogenetic clusters were defined using three complementary criteria: (i) phylogenomic placement as well-supported monophyletic groups in the whole-genome SNP tree, based on UFboot, aBayes, and SH-aLRT branch support values; (ii) genealogical concordance, assessed by the recovery of the same monophyletic groups in phylogenies inferred from individual scaffolds; and (iii) concordance with population structure, evaluated using the variational Bayesian framework implemented in fastSTRUCTURE.

Deviation from the expected 1:1 MAT1-1: MAT1-2 ratio was evaluated independently for each clade. Because several clades contained small numbers of isolates, two-sided exact binomial tests were used as the primary inference framework, with chi-square goodness-of-fit tests reported for comparison when expected counts permitted. Clades with fewer than four isolates were interpreted cautiously, and lack of statistical significance in these cases was attributed to limited power. Associations between phylogenetic distribution and epidemiological variables (source, state, and year) were assessed, in which each clade was contrasted against all other isolates. For each variable, contingency tables were constructed after excluding isolates with unknown values. To accommodate sparse tables and low expected counts, statistical significance was evaluated using permutation-based χ^2^ tests of independence, in which clade labels were randomly permuted to generate empirical null distributions of the chi-square statistic. All tests were two-sided, and results are reported as permutation p-values.

### Copy number variation (CNV) analysis

Initially, each reference genome assembly was split into one FASTA file per contig using Python 3.11 and Biopython 1.81. Contig headers were sanitized to filesystem-safe names, sequences wrapped at 60 bp, and an index (index_contigs.csv) was generated that included the original headers and lengths. For CNV analyses, reads were mapped in a species-specific manner: *S. brasiliensis* isolates were aligned to the *S. brasiliensis* reference genome GCF_000820605.1, whereas *S. schenckii* isolates were aligned to the *S. schenckii* reference genome GCA_000961545.1 using the same methods as described above. To scan for telomeres, the last 10 kb at both ends of each contig were analyzed for the canonical fungal hexamer TTAGGG (and its complementary CCCTAA) using TIDK [44]. The total number of overlapping motif hits and the longest exact tandem run (in base pairs and 6-mer “units”) were recorded. A contig end was considered telomeric if it contained ≥5 exact units and ≥10 motif hits, and contigs with telomeric ends on both sides were labeled as telomer-to-telomere (T2T). Centromere candidates were inferred using a regex-based search for simple tandem arrays (repeat unit k = 2–10 bp, repeated ≥12 times), merging overlaps and annotating coordinates, length, repeat unit/k, distance to each contig end, and local GC% within a ± 5 kb window. The top candidate per contig was flagged as the longest array located ≥20 kb from both ends. Repeats were inferred and masked using RepeatMasker and EDTA [45].

To detect CNVs associated with genomic alterations such as gains and losses, Control-FREEC, a tool that uses a read-depth-based approach, was applied to the realigned BAM files [46]. The following parameters were used: breakpoint threshold = 0.8, ploidy = 1, minimum expected GC content = 0.33, maximum expected GC content = 0.63, read count threshold = 10, telocentromeric value = 0, and a window size of 1,000 base pairs. For each sample, the mean and variance of copy numbers were computed using a 250-bp window to quantify variation in sequencing depth along assembled genomes. To quantify the extent of CNV differentiation between populations, VST (Variance Standardized by T) was calculated, ranging from 0 (no differentiation in CN allele frequencies between populations) to 1 (complete differentiation in CN allele frequencies between populations). CNVs were inferred from read-depth variation using Control-FREEC, and downstream comparisons of CNV content among lineages were performed in R using the *stats* package. To visualize CNV patterns, chromosome-wide CNV heatmaps were generated using the Pandas library within an IPython environment (Python v3.9.13) (Python v3.9.13) [47], showing regions of copy number loss and duplication across individual samples. VST values were also plotted along genomic positions to highlight regions of significant divergence.

### Genome-wide association analysis to identify variant sites associated with resistance and population structure

The R package ‘treeWAS’ (version 1.1) was used to identify genomic sites significantly associated with antifungal drug resistance and population structure/lineage. A maximum likelihood phylogeny was constructed for isolates with known itraconazole minimum inhibitory concentrations (MICs), comprising 66 isolates with 820,230 positions as previously described [26]. Analyses were performed in R version 4.2.3 using a binary phenotype, with an MIC cutoff of 16 to define resistance and a *p-value* cutoff of 0.01 for all three association tests. Significant *p-values* were averaged across the three tests. The same approach was repeated to identify genomic variants significantly associated with population structure, using lineage membership provided as a discrete variable for the phenotype.

Significant GWAS-associated variant sites were annotated by parsing the GFF3 annotation file of the *S. brasiliensis* reference genome RefSeq GCF_000820605.1. Variants were classified according to their genomic position relative to annotated features, including predicted coding regions, introns, intergenic regions, and upstream/downstream regions. For variants associated with predicted coding features, functional information was assigned based on the corresponding RefSeq annotations and closest orthologs. Gene Ontology enrichment analyses were performed using the set of predicted ORFs associated with significant GWAS variants as the query set, with the predicted coding content of the reference genome used as background. Enriched GO terms were summarized and visualized using REVIGO [48].

## Results

### Phylogenetic assessments

For phylogenomic reconstruction, the maximum-likelihood (ML) tree was inferred from a combined dataset comprising 2,274,664 genome-wide SNPs across both *S. brasiliensis* and *S. schenckii*. These variant sites were used to generate a maximum likelihood (ML) tree. As expected, the tree showed clear differentiation between the two species, demonstrating strong branch support and reciprocally monophyletic lineages (Fig 1A). The *S. brasiliensis* tree partition revealed six highly supported clades, each containing closely related isolates. Notably, each of these clades were geographically restricted, indicating divergence was influenced by distance within Brazil.

**Fig 1:**
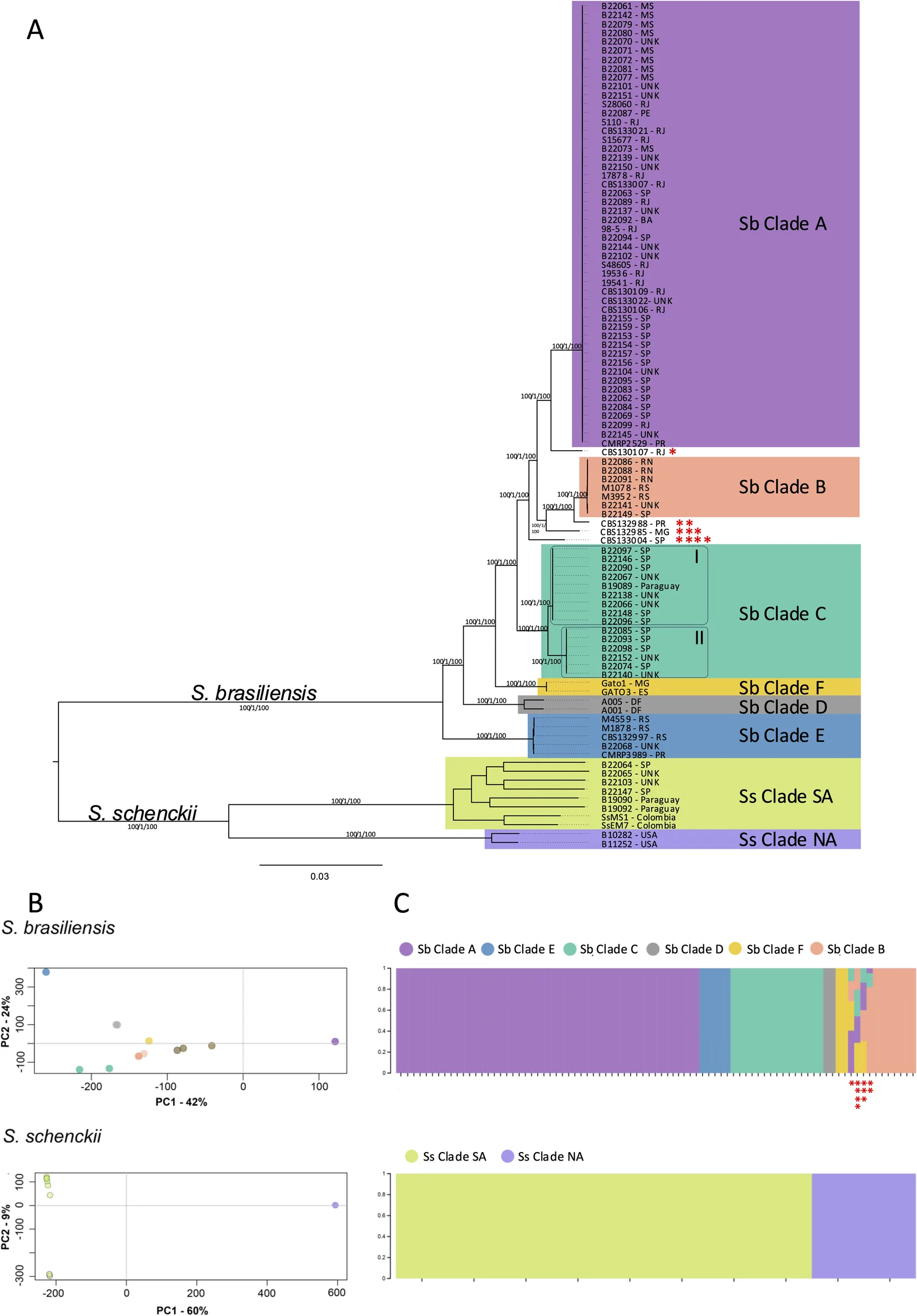
Phylogenomic structure and population stratification of *Sporothrix brasiliensis* and *S. schenckii.* **(A)** Maximum-likelihood (ML) phylogeny inferred from 2,274,664 genome-wide SNPs showing a strong separation between *S. brasiliensis* and *S. schenckii* isolates. Six well-supported clades were recovered within *S. brasiliensis* **(A–F)**, whereas *S. schenckii* segregated into two major phylogeographic lineages corresponding to South America (SA) and North America (NA). Node supports are based on ultrafast bootstrap (UFBoot) and aBayes values from IQ-TREE2. **(B)** Principal component analysis (PCA) of 94 isolates, illustrating clustering according to species and population. *S. brasiliensis* clades cluster into discrete genetic groups, while *S. schenckii* shows clear separation between North and South American isolates. **(C)** Ancestry inference analysis using FastStructure and ADMIXTURE showing K = 6 populations in *S. brasiliensis* and K = 2 in *S. schenckii*. Each vertical bar represents one genome, and colors correspond to ancestral population membership. Four *S. brasiliensis* isolates (CBS130107, CBS132988, CBS132985, and CBS133004) exhibit mixed ancestry, consistent with admixture or incomplete lineage sorting.

Population genetic analyses restricted to the *S. brasiliensis* dataset yielded consistent results when performed using the 610,242 species-specific SNPs. In *S. brasiliensis*, PCA revealed that a substantial portion of the genetic variation was captured by the first principal component (PC1), which accounted for 42% of the total variance (Fig 1B). PC1 discriminates between Clades A, B, C, D and F. PC2, which explains 24% of the variance, distinguished Clades D and E from the remaining isolates. FastStructure analysis also indicated that the genetic variance in S*. brasiliensis* is best explained by six clusters (Fig 1C, S2 Table).

Clade A in *S. brasiliensis* (Sb Clade A), includes isolates from Rio de Janeiro, São Paulo, Pernambuco, Paraná, Bahia and Mato Grosso do Sul and is dominant. This clade was previously classified as the RJ population or H1 based on MLST data [11,18]. The group is comprised of samples from both human (n = 36) and cat (n = 14), with the majority collected from Brazil between 1998–2021, coinciding with the sporotrichosis epidemics. Clade B (Sb Clade B; 7 isolates), which includes isolates from Rio Grande do Norte, São Paulo, and Rio Grande do Sul (designated as clade RS or H3), emerged no earlier than 2012 [11,18]. Clade C (Sb Clade C) is predominantly composed of human-derived isolates from São Paulo, with a single veterinary isolate (from a cat) identified in Paraguay. Two strongly supported subclades are observed within this clade. The cat was brought to Paraguay by its owners from Guarulhos, SP. Clade D (Sb Clade D) consists of veterinary-derived isolates from the Federal District of Brazil, consistent with MLST (H6-7) and previous genome-typing findings [6,11,18]. Clade E (Sb Clade E) contains isolates from Rio Grande do Sul and Paraná, representing a second cryptic *S. brasiliensis* lineage emerging in southern Brazil. Clade F (Sb Clade F) represents a genetic southeastern Brazilian lineage composed of two cat-derived isolates from Minas Gerais and Espírito Santo and previously genotyped as H2 via MLST data [18]. Isolates CBS130107 (Rio de Janeiro), CBS132988 (Paraná), CBS132985 (Minas Gerais), CBS133004 (São Paulo), are positioned between strongly supported clades, a pattern seen in individual contig genealogies (S1 Fig) which may reflect unsampled intermediate lineages, or potential cases of admixture or incomplete lineage sorting (Fig 1A).

For *S. schenckii*, species-restricted population genetic analyses based on 1,474,627 SNPs produced concordant results across the maximum-likelihood phylogeny (Fig 1A), PCA (Fig 1B), and fastStructure analyses (Fig 1C; S2 Table), supporting the presence of two major populations: one restricted to North America and the other to South America. While PC1 accounted for 60% of the genetic variation, clearly separating the South American (Ss Clade SA) and the North American (Ss Clade NA) groups, distinguishing isolates by continental origin (Fig 1B). PC2, which accounted for 9% of the variance, captured finer-scale genomic differentiation among South American isolates, including those from Brazil, Paraguay, and Colombia. Together, these axes reveal a strong pattern of population structure and geographic isolation within each of the two species of *Sporothrix* included in this study.

### Distribution of epidemiological variables across clades

Mating-type frequencies were evaluated within each clade against a 1:1 expectation using both exact binomial and chi-square goodness-of-fit tests. All clades were fixed for a single mating type, except Clade D (from Federal District), which was the only population containing both MAT1–1 and MAT1–2 (1:1) and therefore showed no deviation from the expected ratio (binomial p = 1.0; χ^2^ p = 1.0). In contrast, Sb Clade A was completely MAT1–2 (49/49) and showed the strongest distortion (binomial p = 3.55 × 10^−15^; χ^2^ p = 2.56 × 10^−12^), while Sb Clade C-I and Sb Clade B were entirely MAT1–1 (10/10 and 7/7, respectively) and Sb Clade C-II entirely MAT1–2 (6/6), consistent with significant departures from 1:1 by both frameworks (exact p ≤ 3.13 × 10^−2^). It is worth nothing that Sb Clade C-I contains only isolates with the MAT1–1 idiomorph in the present dataset, whereas Sb Clade C-II is populated by the MAT1–2 allele, suggesting two independent clonal expansions that occurred between 2017 and 2021 within São Paulo state of Brazil. Sb Clades E and F were both fixed for MAT1–1 but did not show statistically significant deviation from the expected 1:1 mating-type due to low sample size. Collectively, these results indicate pervasive mating-type fixation across clades, with Sb Clade D as the only exception retaining both mating types (Table 1).

**Table 1:** Distribution of mating-type idiomorphs (MAT1-1 and MAT1-2) across *Sporothrix brasiliensis* clades. For each clade, the number of isolates (n) carrying MAT1-1 or MAT1-2 is shown. Deviations from the expected 1:1 mating-type ratio were tested using binomial and χ^2^ goodness-of-fit tests.

Clade	n	*MAT1–1*	*MAT1–2*	p (binomial)	χ^2^	p (χ^2^)	min expected
Sb Clade A	49	0	49	3.55e-15	49.00	2.56e-12	24.5
Sb Clade B	7	7	0	1.56e-02	7.00	8.15e-03	3.5
Sb Clade C-I	10	10	0	1.95e-03	10.00	1.57e-03	5.0
Sb Clade C-II	6	0	6	3.13e-02	6.00	1.43e-02	3.0
Sb Clade D	2	1	1	1.00	0.00	1.00	1.0
Sb Clade E	5	5	0	6.25e-02	5.00	2.53e-02	2.5
Sb Clade F	2	2	0	5.00e-01	2.00	1.57e-01	1.0

Associations between genetic clades and epidemiological variables were evaluated by testing clade-specific distributions of source, geographic origin, and sampling year against the overall dataset after exclusion of admixed isolates. To accommodate sparse data and unequal sampling, statistical significance was assessed using permutation-based chi-square tests, and inference was restricted to clades represented by at least four isolates. Under this framework, geographic state of origin showed consistent evidence of association with clade structure. In particular, Clades A, B, and E exhibited non-random state distributions relative to the global isolate set (permutation *p* = 2.0 × 10^−4^, 1.0 × 10^−3^, and 0.012, respectively), indicating pronounced geographic structuring. In contrast, no significant associations were detected with sampling year (all permutation *p* ≥ 0.24) or source (all permutation *p* ≥ 0.31) among clades meeting the minimum sample-size threshold. Together, these results indicate that geographic origin is the dominant epidemiological correlate of clade structure, whereas host/source and temporal variables do not show detectable clade-specific enrichment in the present dataset.

### Genome-wide CNV architecture in *S. brasiliensis* and *S. schenckii*

Next, we investigated the presence of structural variants within *S. brasiliensis and S. schenckii*. Telomeric ends and centromeres were identified in both genomes (GCF_000820605.1 and GCA_000961545.1). For downstream analysis of CNV, the distributions of telomeric run units and candidate centromere lengths were masked. The genome-wide CNV profiles of *S. brasiliensis* and *S. schenckii* revealed a predominant trend toward genomic losses over gains in both species, *S. schenckii* (S2 Fig) and *S. brasiliensis* (S3 Fig). Both species showed heterogeneous CNV landscapes that differed between them. In *S. brasiliensis*, CNVs formed well-delimited blocks alternating between amplification and loss across hundreds of kilobases (Fig 2A). The largest variation was observed on contig NW_024467135.1, where duplications and deletions frequently flanked sub-telomeric regions enriched in repetitive sequences. High VST (Variance based FST) values indicated regions where CNVs varied between populations. Contigs NW_024467133.1, and NW_024467134.1 displayed duplication hotspots that harbored a large portion of the detected duplications (Fig 2A). Overall predicted coding sequences losses were predominant, suggesting large-scale deletions or pseudogenization rather than tandem expansion in *S. brasiliensis*.

**Fig 2:**
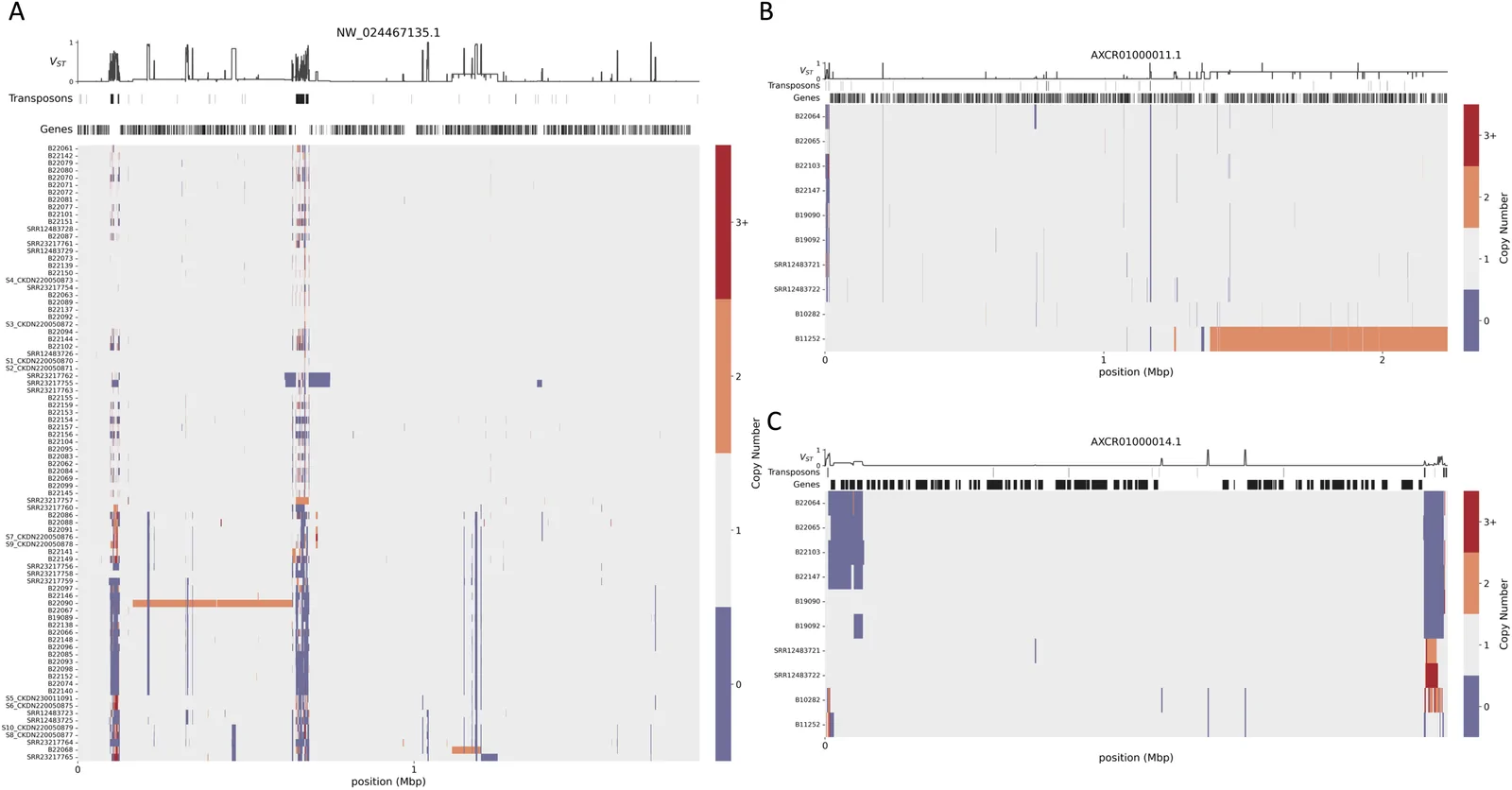
Copy number variation across representative contigs of *Sporothrix brasiliensis* and *S. schenckii.* **(A)** *S. brasiliensis* contig NW_024467135.1 showing large CNV blocks with alternating loss (blue) and duplication (red) events. **(B)** *S. schenckii* contig AXCR01000011.1, exhibiting a continuous high-amplitude duplication region exceeding 100 kb. **(C)** *S. schenckii* contig AXCR01000014.1, marked by irregular interspersed duplication and deletion segments generating a complex CNV landscape.

In contrast, the CNV landscape *S. schenckii* was characterized by multiple interspersed duplications and deletions (Fig 2B-2C). Contig AXCR01000011.1 showed a large duplication block exceeding 100 kb, particularly prominent in strain B11252, and was interrupted by sharp deletion valleys resulting in a mosaic pattern of gain and loss. Contig AXCR01000014.1 displayed loss events that were polymorphic among strains (B22064, B22065, B22103, B22147, B19092 and B11252). Genome-wide, the *S. schenckii* genome had 36 CNV hotspots, predominantly concentrated on AXCR01000011.1 (16 hotspot windows) and AXCR01000006.1 (6 windows), with additional focal peaks observed on contigs AXCR01000001.1, AXCR 01000007.1, AXCR 01000010.1, AXCR 01000012.1, and AXCR 01000014.1.

Next, the predicted coding sequences content of the identified CNVs was characterized. A total of 158 unique CNV-affected predicted ORFs were identified in *S. brasiliensis* (60 gains and 98 losses), which corresponds to approximately 1.7% of the total predicted ORF content of the reference genome (strain 5110). In *S. schenckii*, 88 predicted coding sequences (54 gains and 34 losses) were affected, representing about 0.5% of the reference genome (strain 1099-18). The extent of structural variation not only differed between species but also varied within each species (Fig 3).

**Fig 3:**
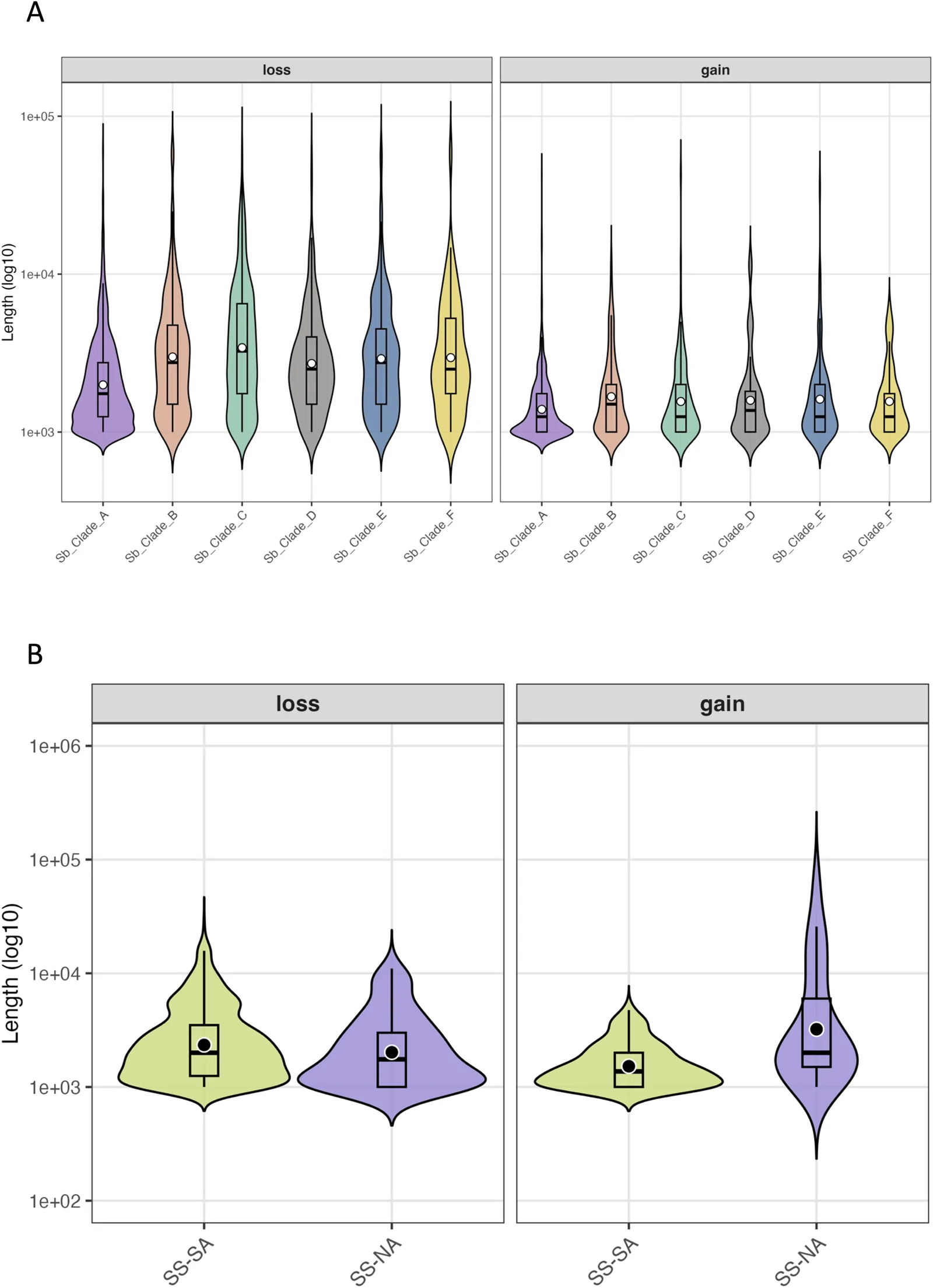
Copy number variation (CNV) analysis in *Sporothrix* species. A Violin plot illustrating distribution of copy number variations characterized by loss and gain across various clades in **A)** *Sporothrix brasiliensis* (Sb Clade A, Sb Clade B, Sb Clade C, Sb Clade D, Sb Clade E, and Sb Clade F) and **B)** *S.schenckii* (South American Clade, North American Clade). The y-axis represents the length of CNVs on a logarithmic scale (log10), indicating the magnitude of the variations. The x-axis categorizes the data into different clades, allowing for a comparison of CNV patterns among them.

The CNV burden displayed considerable variability within both species, following distinct population-level patterns. In *S. brasiliensis*, most CNVs were isolate-specific, with minimal overlap between individuals. Clade Sb A carried the largest number of CNVs relative to the reference genome (22 gains, 63 losses), followed by Clade Sb C (20 gains, 41 losses) and Clade Sb E (9 gains, 34 losses). Clade F showed the lowest count, with only four loss events (Fig 3A). In *S. schenckii*, CNV dynamics differed between the two lineages. North American (NA) isolates showed more gains than losses relative to the reference genome (54 gains vs 1 loss; approximately 2.12 Mb total CNV span). On the other hand, South American (SA) isolates showed only deletions with respect to the reference (0 gains vs 57 losses; approximately 0.87 Mb) (Fig 3B). No predicted ORFs displayed opposing CNV states (gain vs loss) between the two lineages, indicating non-overlapping targets of structural variation.

When comparing the functional repertoires of *Sporothrix brasiliensis* and *S. schenckii*, the two species showed dissimilar trends in predicted coding sequences gain and loss driven by CNV events. In *S. brasiliensis*, CNV-associated gains are mainly associated with molecular functions related to protein kinase activity, phosphotransferase activity, ATP binding, and proteins associated with microtubule trafficking (S4 Fig, S3 Table). Conversely, the losses in *S. brasiliensis* primarily involve ORFs encoding ribosomal proteins, structural constituents of translation, oxidoreductases, and enzymes involved in amino acid and lipid metabolism. (S4 Fig, S3 Table). For *S. schenckii,* the gains were focused on transporters, ion-binding proteins, and oxidoreductase enzymes. The losses included kinases and regulatory proteins, (S5 Fig, S4 Table). These findings indicate that the genes and gene categories affected by CNVs differ between both *Sporothrix* species.

### Genome-wide associations reveal multifactorial basis of itraconazole resistance in *Sporothrix brasiliensis*

Finally, we conducted a genome-wide association study (GWAS) to identify variants associated with resistance to itraconazole. While this approach does not provide functional evidence of the involvement of alleles in the trait, the results can reveal the genetic architecture of the trait, and provide candidate genes to validate. A total of 81 SNPs that were identified genome-wide as significantly associated with the resistance trait (p < 1 x 10^ 5); (Fig 4; S5 Table). The analysis revealed single scattered SNPs rather than distinct genomic regions linked to the trait. Significant variants were distributed across multiple scaffolds. Among these, 29 SNPs mapped to coding regions, including 14 synonymous (SYN) and 15 nonsynonymous (NSY) substitutions, 50 to intergenic intervals (including upstream and downstream regulatory regions), and two in intronic region. The coding variants comprised of both nonsynonymous and synonymous substitutions (S5 Table). ORF-level mapping highlighted several candidate genes, including SPBR_02168 (2 sites) and SPBR_00021, SPBR_00237, SPBR_00359, SPBR_00667, SPBR_02233, SPBR_02392, SPBR_02543, SPBR_03592, SPBR_03640, SPBR_04097, SPBR_05759, SPBR_05765, SPBR_06183, and SPBR_06651 (1 site each). All genomic variants were subsequently annotated with the closest orthologs (S6 Table). None of the alleles have been previously implicated in azole resistance. Gene Ontology (GO) analyses indicated that this dataset is enriched for ABC transporters (ORFs = 16, Fold enrichment = 6.9; Enrichment FDR = 6.4 × 10^−11^), mismatch repair (ORFs = 9, Fold enrichment = 3.5; Enrichment FDR = 5.6 × 10^−3^), and DNA repair (ORFs = 12,Fold enrichment = 3.0; Enrichment FDR = 4.0 × 10^−3^).

**Fig 4:**
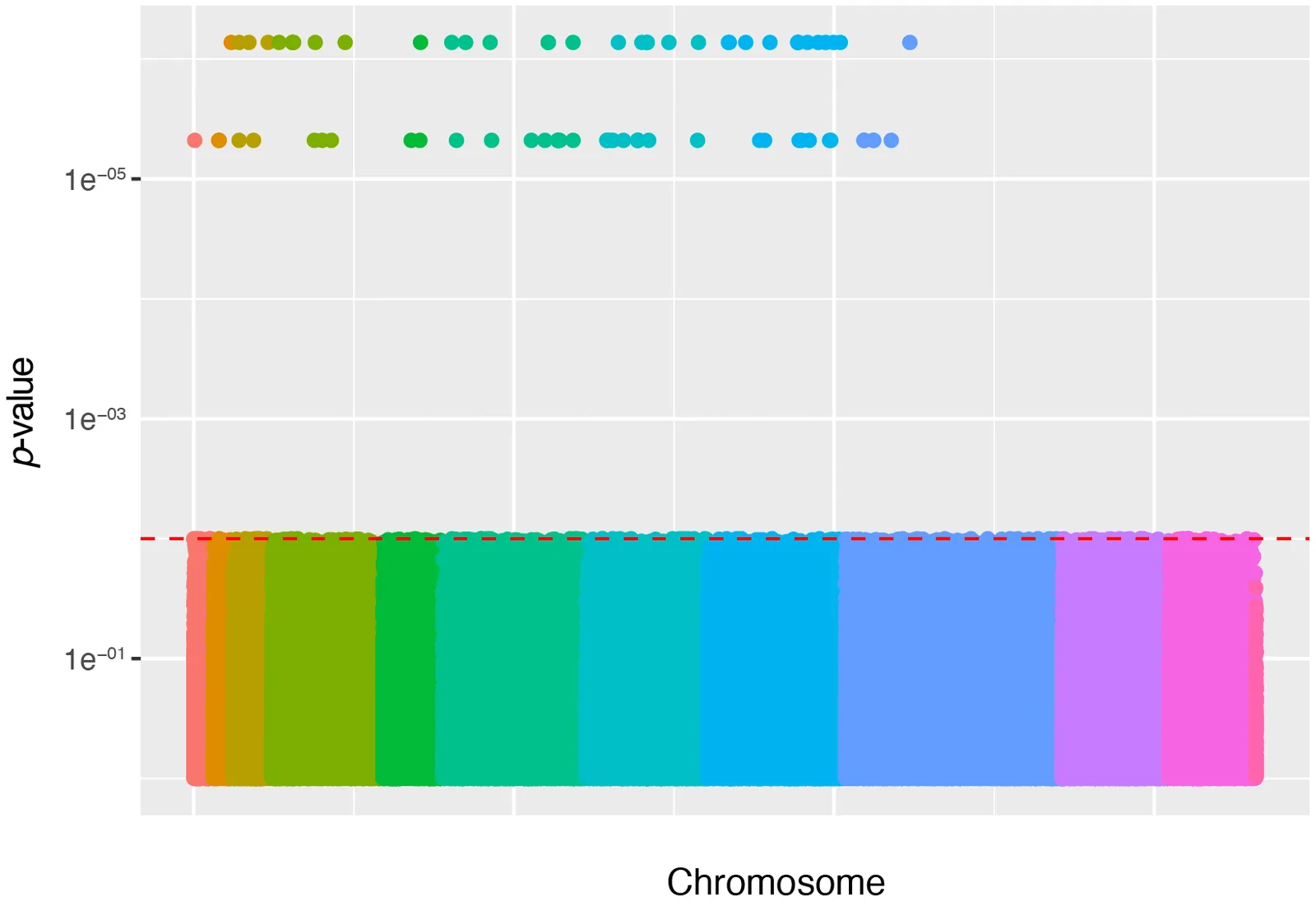
Genome-wide association analysis of itraconazole resistance in *Sporothrix brasiliensis.* Manhattan plot depicting the genome-wide distribution of SNPs associated with itraconazole resistance across all contigs. Each point represents an individual SNP, and colors alternate by chromosome for visual distinction. The y-axis shows the –log_10_(*p*-value) of association under a binary resistance phenotype (MIC ≥ 8 µg/mL).

## Discussion

The magnitude and types of genetic variation in fungal pathogens represent an active area of research [49]. Deciphering this diversity is essential to identify the signatures of natural selection acting on pathogenic traits, including virulence, host adaptation, and antifungal tolerance. This question is particularly pressing for *Sporothrix*, a major One Health pathogen, where clonal expansion and geographic spread are reshaping disease burden but the evolutionary drivers of emergence are still unclear [2]. In this study, nucleotide and structural variation of *S. brasiliensis* and *S. schenckii* across the American continent were quantified. In this piece, we combine the power of SNP-based phylogenomics, CNV profiling, and GWAS, to uncover patterns of population structure, structural variation, and antifungal resistance in fungal emerging pathogens. Our analyses indicate that *S. schenckii* exhibits substantially greater genomic diversity than *S. brasiliensis* [4,17]. The deep phylogeographic split between the North and South American *S. schenckii* lineages, coupled with strong population structure suggests long-term geographic isolation (Fig 1). In contrast, *S. brasiliensis* clades are dominated by low diversity and uneven mating type distribution, which is indicative of rapid clonal expansion from a recent common ancestor [4,11,50]. The predominance of Sb Clade A across multiple and distant Brazilian states and hosts underscores the widespread dissemination of a successful epidemic genotype, while the existence of geographically restricted lineages (e.g., Sb Clades C, D and E) reflects less sampled lineages that require further investigation [4,11,19,51]. Across *S. brasiliensis* populations, mating-type structure was highly skewed, with most clades showing complete fixation for a single idiomorph (Table 1), consistent with predominantly clonal propagation. Notably, the Federal District (Sb Clade D) was the only population in which both *MAT1-1* and *MAT1-2* were detected at equal frequency (1:1), indicating the coexistence of both mating types in this region [17]. The dominance of a single mating type across geographic clades supports multiple independent clonal emergences and dissemination events, consistent with founder effects and rapid regional spread driven largely by clonal transmission [4,11,19].

Evidence of admixed genotypes in certain *S. brasiliensis* isolates challenges the classical view of strict clonality and suggests the possibility of occasional genetic exchange [52,53]. Although mosaic ancestry appears uncommon, its presence raises important questions about the degree of connectivity among *S. brasiliensis* lineages across their broad geographic distribution (Fig 1) [54]. Similar signals of recombination and intraspecific horizontal transfer have been reported in other fungal pathogens lacking a known sexual stage, indicating that genetic exchange can occur even when classical sexual reproduction has not been observed [55,56]. Nonetheless, the underlying mechanism(s) generating these patterns in pathogenic *Sporothrix* (sexual, parasexual, or driven by other processes) remain unresolved despite decades of investigation [11,57].

Our CNV analyses revealed different genomic patterns in the two *Sporothrix* species studied. *S. brasiliensis* showed a larger number of genes with evidence of CNVs (158 versus 88 in *S. schenckii*), with most events representing deletions rather than duplications. These CNVs were more prevalent in the subtelomeric regions (Fig 2), which are recognized hotspots for adaptive evolution in pathogenic fungi [58], and preferentially affected genes involved in translation, metabolism, and cell wall biosynthesis. Conversely, CNV gains in *S. brasiliensis* were concentrated in genes related to kinase signaling, Golgi organization, and vesicle trafficking. *S. schenckii*, on the other hand, showed gains in transporters, oxidoreductases, and metabolic enzymes, while losses were primarily in regulatory or signaling elements. The underlying reasons of these differences might be ecological, demographic, or related to variations in sample size. A more systematic sampling approach, coupled with highly contiguous assemblies of both species, will be necessary to investigate these potential causes further.

The results presented here provide an initial approximation for identifying the genetic basis of azole resistance in *S. brasiliensis*. We identified 81 sites associated with high resistance to fluconazole scattered throughout the genome (Fig 4). Notably, none of the alleles significantly associated with this trait have been previously implicated with azole resistance in any fungi. Although the current sample size and the limited availability of standardized MIC data reduce the statistical power to detect variants with small effect sizes, the identified associations provide the first genome-wide catalogue of candidate loci potentially involved in azole susceptibility in *Sporothrix*. As with genome-wide association studies in other organisms, these statistically supported associations are intended to generate biological hypotheses that require subsequent validation through functional genetic approaches, including targeted mutagenesis, allele replacement, transcriptomic analyses, or gene expression studies. Nevertheless, the identification of these candidate loci represents an essential first step toward elucidating the molecular basis of azole resistance in *Sporothrix*, providing a prioritized set of targets for future mechanistic investigations. These results should therefore be interpreted as a foundation for future validation functional studies using genetic modifications rather than definitive evidence of causal resistance mechanisms.

Our study has other caveats that warrant attention. The geographic sampling of *Sporothrix* remains uneven, with many endemic regions across the geographic range of the genus either underrepresented or unsampled. This uneven sampling may bias population-level inferences and underestimating the level of genetic diversity [59]. A coordinated network of genomic epidemiology is needed to understand the emergence of these pathogens [2]. Because CNV inference was based on read-depth variation relative to a reference genome, the results may be influenced by reference bias. This is particularly relevant given the population structure observed in our dataset, which included at least six *S. brasiliensis* populations and two *S. schenckii* populations. Sequence divergence among these lineages may affect read-mapping efficiency and, consequently, the accuracy of CNV estimates. Long-read sequencing and chromosome-level assemblies from representative isolates will be needed to validate these CNV patterns and more accurately resolve structural variation across *Sporothrix* lineages (see [60]).

Collectively, this study establishes the first comprehensive population genomic resource for pathogenic *Sporothrix* spp. and provides the first genome-wide catalogue of candidate genetic determinants associated with antifungal susceptibility [13]. Beyond the biological insights presented here, the analytical framework developed in this study is readily transferable to larger collections and other medically important fungal pathogens, creating a foundation for future functional studies, genomic surveillance, and the discovery of molecular mechanisms underlying antifungal resistance [2,61].

## Supporting information

S1 Fig Scaffold-based phylogenetic analyses reveal mosaic topologies within *Sporothrix brasiliensis.* Maximum-likelihood (ML) phylogenies reconstructed from individual scaffolds of *S. brasiliensis* and *S. schenckii* show overall concordance with the whole-genome SNP tree but highlight local topological shifts among several *S. brasiliensis* isolates. Isolates CBS130107, CBS133004, CBS132985, and CBS132988 exhibit variable placement across scaffolds. Branch support values are derived from ultrafast bootstrap (UFBoot) and aBayes metrics.
(PDF)

S2 Fig Chromosome-level distribution of copy number variants (CNVs) in *Sporothrix brasiliensis.* Bar plots showing CNV counts for each chromosome across individual isolates of *S. brasiliensis*. Red bars represent copy number gains, and blue bars represent losses.
(TIF)

S3 Fig Chromosome-level distribution of copy number variants (CNVs) in *Sporothrix schenckii.* Bar plots showing CNV counts for each chromosome across individual isolates of *S. schenckii*. Red bars represent copy number gains, and blue bars represent losses.
(TIF)

S4 Fig Cytoscape bubble charts summarizing GO enrichment for CNV-associated genes in *Sporothrix brasiliensis.* Panels: (A) Biological Process—gains; (B) Molecular Function—gains; (C) Biological Process—losses; (D) Molecular Function—losses. Each bubble represents a GO term; bubble color encodes the Value provided with each GO term in the dataset, and bubble size reflects the term’s LogSize. Terms were summarized and visualized in Cytoscape to highlight functional themes among CNV gains versus losses.
(PDF)

S5 Fig Cytoscape bubble charts summarizing GO enrichment for CNV-associated genes in *Sporothrix schenkii.* Panels: (A) Biological Process—gains; (B) Molecular Function—gains; (C) Biological Process—losses; (D) Molecular Function—losses. Each bubble represents a GO term; bubble color encodes the Value provided with each GO term in the dataset, and bubble size reflects the term’s LogSize. Terms were summarized and visualized in Cytoscape to highlight functional themes among CNV gains versus losses.
(PDF)

S1 Table Metadata and antifungal susceptibility profiles of Sporothrix brasiliensis isolates included in this study. The table summarizes isolate identifiers, year of collection, source (human/animal/environment), geographic origin (country, state, city), population assignment (clade), inferred mating-type idiomorph (MAT1–1 or MAT1–2), and minimum inhibitory concentrations (MICs) for itraconazole (ITR), amphotericin B (AMB), and terbinafine (TER), when available. Sequence read archive (SRA) accession numbers and original references are provided for publicly available genomes, alongside newly generated isolates reported here. “UN” indicates unknown metadata fields, and isolates labeled as “Admixed” represent genomes with mosaic ancestry based on population structure analyses.
(XLSX)

S2 Table Population-structure model evaluation using fastSTRUCTURE and ADMIXTURE. Marginal likelihood, cross-validation values, and standard deviations are shown for K = 1–12 in *Sporothrix brasiliensis* and *Sporothrix schenckii*. The model components supported K = 6 for *S. brasiliensis* and K = 2 for *S. schenckii*, consistent with the major phylogenetic groups recovered in the SNP-based analyses.
(DOCX)

S3 Table Summary of CNV-associated functional enrichments in *S. brasiliensis.* (XLSX)

S4 Table Summary of CNV-associated functional enrichments in *S. schenckii.* (XLSX)

S5 Table Genetic variants associated with antifungal susceptibility in *Sporothrix brasiliensis.* The table lists SNPs and genomic variants identified across coding and intergenic regions, including variant name, locus, scaffold/chromosome, genomic position, functional class, affected gene, predicted amino acid change, and association p-values for individual antifungal comparisons and the overall test. Synonymous and nonsynonymous coding variants are indicated as SYN and NSY, respectively; “NA” indicates comparisons in which the variant was not retained or tested.
(XLSX)

S6 Table Functional annotation of *Sporothrix brasiliensis* genes harboring antifungal-associated variants. The table summarizes gene identifiers, genomic coordinates, predicted product descriptions, orthology and paralogy information, nonsynonymous/synonymous SNP ratios, and conserved domain annotations based on PFam and InterPro. These annotations provide functional context for genes containing variants associated with antifungal susceptibility profiles.
(XLSX)
