# Supplementary material for: Genome variation of Sporothrix schenckii and Sporothrix brasiliensis
Source: PLoS Negl Trop Dis. 2026 Sep 21;20(9):e0014203. doi: 10.1371/journal.pntd.0014203 (PMC13641699; doi:10.1371/journal.pntd.0014203)
Supplement: Supplementary file 1 [file pntd.0014203.s001.pdf]

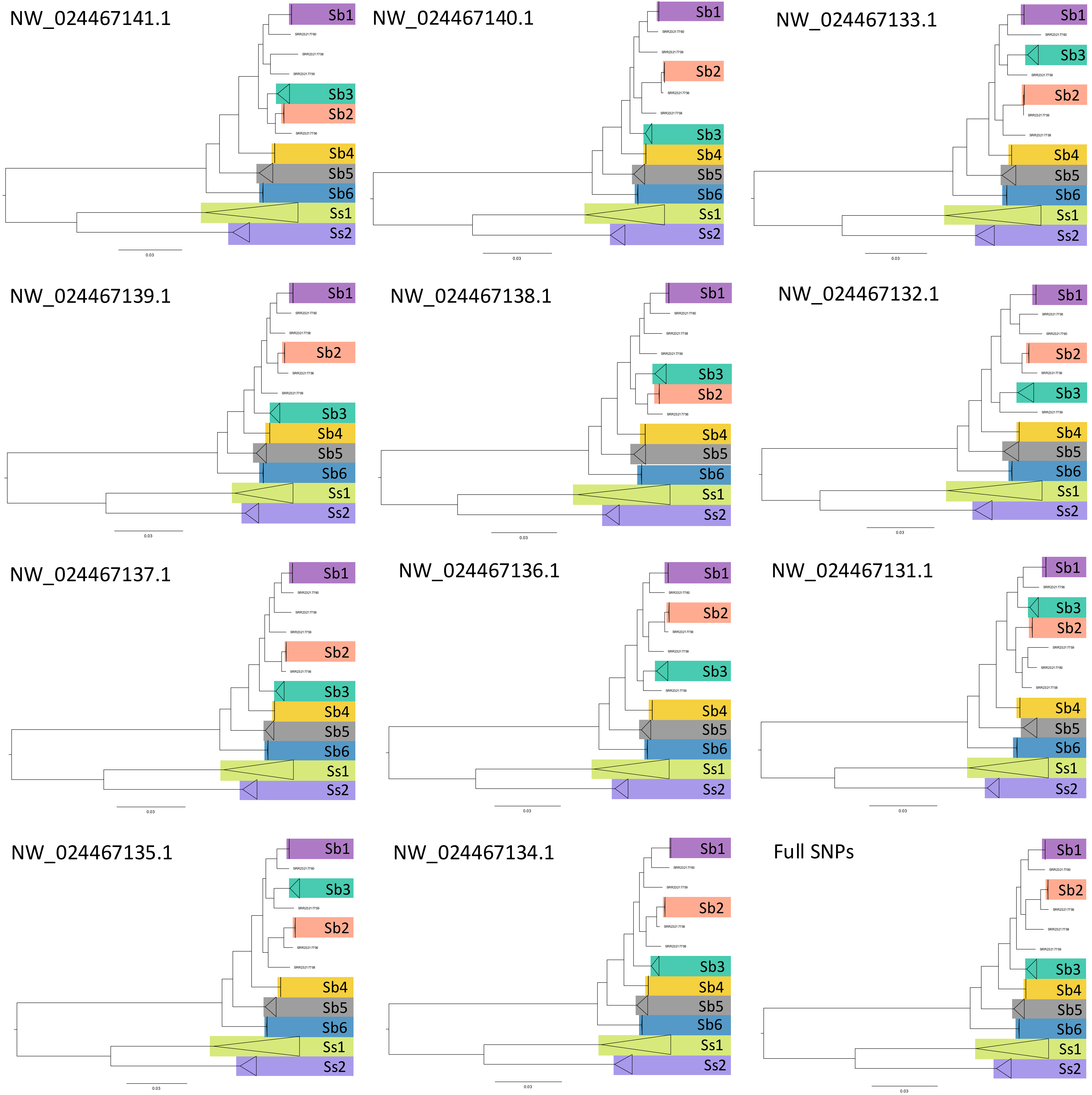
NW_024467141.1
Sb1
Sb3
Sb2
Sb4
Sb5
Sb6
Ss1
Ss2
0.03
NW_024467140.1
Sb1
Sb2
Sb3
Sb4
Sb5
Sb6
Ss1
Ss2
0.03
NW_024467133.1
Sb1
Sb3
Sb2
Sb4
Sb5
Sb6
Ss1
Ss2
0.03
NW_024467139.1
Sb1
Sb2
Sb3
Sb4
Sb5
Sb6
Ss1
Ss2
0.03
NW_024467138.1
Sb1
Sb3
Sb2
Sb4
Sb5
Sb6
Ss1
Ss2
0.03
NW_024467132.1
Sb1
Sb2
Sb3
Sb4
Sb5
Sb6
Ss1
Ss2
0.03
NW_024467137.1
Sb1
Sb2
Sb3
Sb4
Sb5
Sb6
Ss1
Ss2
0.03
NW_024467136.1
Sb1
Sb2
Sb3
Sb4
Sb5
Sb6
Ss1
Ss2
0.03
NW_024467131.1
Sb1
Sb3
Sb2
Sb4
Sb5
Sb6
Ss1
Ss2
0.03
NW_024467135.1
Sb1
Sb3
Sb2
Sb4
Sb5
Sb6
Ss1
Ss2
0.03
NW_024467134.1
Sb1
Sb2
Sb3
Sb4
Sb5
Sb6
Ss1
Ss2
0.03
Full SNPs
Sb1
Sb2
Sb3
Sb4
Sb5
Sb6
Ss1
Ss2
0.03
