# Supplementary material for: Genome variation of Sporothrix schenckii and Sporothrix brasiliensis
Source: PLoS Negl Trop Dis. 2026 Sep 21;20(9):e0014203. doi: 10.1371/journal.pntd.0014203 (PMC13641699; doi:10.1371/journal.pntd.0014203)
Supplement: Supplementary file 2 [file pntd.0014203.s004.pdf]

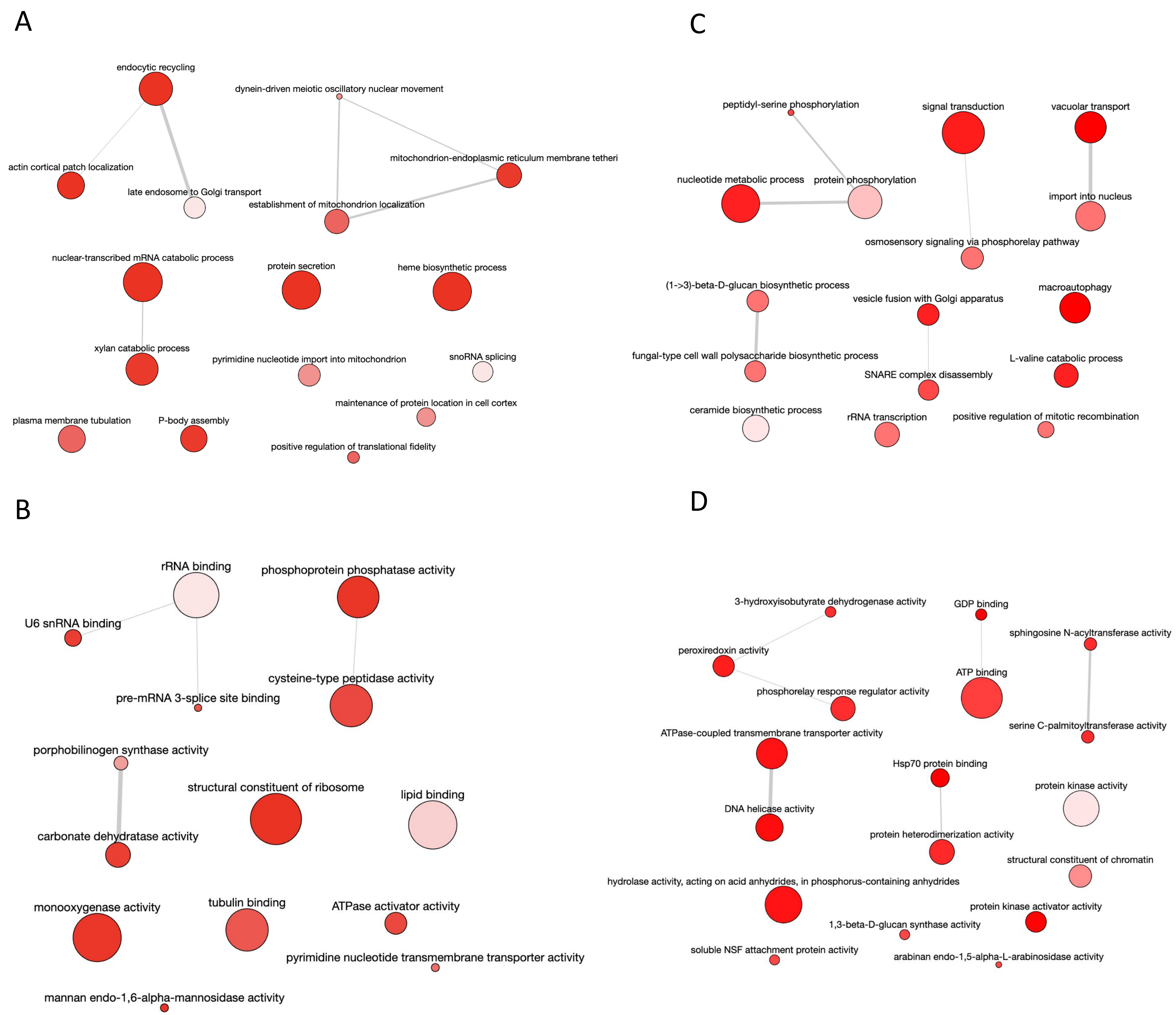

A
endocytic recycling
dynein-driven meiotic oscillatory nuclear movement
actin cortical patch localization
mitochondrion-endoplasmic reticulum membrane tetheri
late endosome to Golgi transport
establishment of mitochondrion localization
nuclear-transcribed mRNA catabolic process
protein secretion
heme biosynthetic process
xylan catabolic process
pyrimidine nucleotide import into mitochondrion
snoRNA splicing
maintenance of protein location in cell cortex
plasma membrane tubulation
P-body assembly
positive regulation of translational fidelity
B
rRNA binding
phosphoprotein phosphatase activity
U6 snRNA binding
cysteine-type peptidase activity
pre-mRNA 3-splice site binding
porphobilinogen synthase activity
structural constituent of ribosome
lipid binding
carbonate dehydratase activity
monooxygenase activity
tubulin binding
ATPase activator activity
pyrimidine nucleotide transmembrane transporter activity
mannan endo-1,6-alpha-mannosidase activity
C
peptidyl-serine phosphorylation
signal transduction
vacuolar transport
nucleotide metabolic process
protein phosphorylation
import into nucleus
osmosensory signaling via phosphorelay pathway
(1->3)-beta-D-glucan biosynthetic process
vesicle fusion with Golgi apparatus
macroautophagy
fungal-type cell wall polysaccharide biosynthetic process
L-valine catabolic process
SNARE complex disassembly
ceramide biosynthetic process
rRNA transcription
positive regulation of mitotic recombination
D
3-hydroxyisobutyrate dehydrogenase activity
GDP binding
sphingosine N-acyltransferase activity
peroxiredoxin activity
ATP binding
phosphorelay response regulator activity
serine C-palmitoyltransferase activity
ATPase-coupled transmembrane transporter activity
Hsp70 protein binding
protein kinase activity
DNA helicase activity
protein heterodimerization activity
structural constituent of chromatin
hydrolase activity, acting on acid anhydrides, in phosphorus-containing anhydrides
protein kinase activator activity
1,3-beta-D-glucan synthase activity
soluble NSF attachment protein activity
arabinan endo-1,5-alpha-L-arabinosidase activity
