# Supplementary material for: Genome variation of Sporothrix schenckii and Sporothrix brasiliensis
Source: PLoS Negl Trop Dis. 2026 Sep 21;20(9):e0014203. doi: 10.1371/journal.pntd.0014203 (PMC13641699; doi:10.1371/journal.pntd.0014203)
Supplement: Supplementary file 3 [file pntd.0014203.s005.pdf]

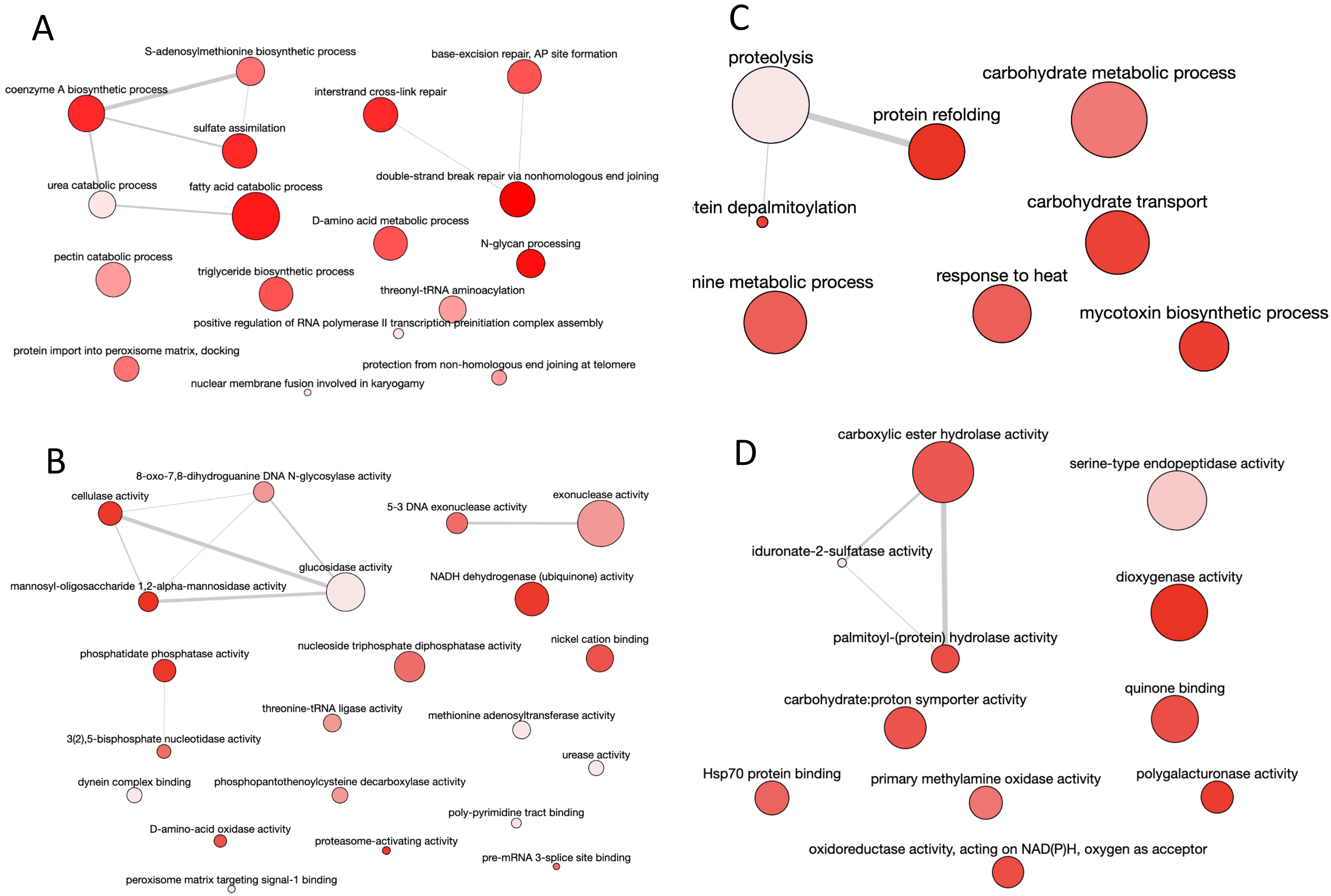
A
S-adenosylmethionine biosynthetic process
base-excision repair, AP site formation
coenzyme A biosynthetic process
interstrand cross-link repair
sulfate assimilation
double-strand break repair via nonhomologous end joining
urea catabolic process
fatty acid catabolic process
D-amino acid metabolic process
N-glycan processing
pectin catabolic process
triglyceride biosynthetic process
threonyl-tRNA aminoacylation
positive regulation of RNA polymerase II transcription preinitiation complex assembly
protein import into peroxisome matrix, docking
protection from non-homologous end joining at telomere
nuclear membrane fusion involved in karyogamy
B
8-oxo-7,8-dihydroguanine DNA N-glycosylase activity
cellulase activity
exonuclease activity
5-3 DNA exonuclease activity
glucosidase activity
mannosyl-oligosaccharide 1,2-alpha-mannosidase activity
NADH dehydrogenase (ubiquinone) activity
nickel cation binding
phosphatidate phosphatase activity
nucleoside triphosphate diphosphatase activity
threonine-tRNA ligase activity
methionine adenosyltransferase activity
3(2),5-bisphosphate nucleotidase activity
urease activity
dynein complex binding
phosphopantothenoylcysteine decarboxylase activity
poly-pyrimidine tract binding
D-amino-acid oxidase activity
proteasome-activating activity
pre-mRNA 3-splice site binding
peroxisome matrix targeting signal-1 binding
C
proteolysis
carbohydrate metabolic process
protein refolding
tein depalmitoylation
carbohydrate transport
nine metabolic process
response to heat
mycotoxin biosynthetic process
D
carboxylic ester hydrolase activity
serine-type endopeptidase activity
iduronate-2-sulfatase activity
dioxygenase activity
palmitoyl-(protein) hydrolase activity
carbohydrate:proton symporter activity
quinone binding
Hsp70 protein binding
primary methylamine oxidase activity
polygalacturonase activity
oxidoreductase activity, acting on NAD(P)H, oxygen as acceptor
