# Supplementary material for: Genome variation of Sporothrix schenckii and Sporothrix brasiliensis
Source: PLoS Negl Trop Dis. 2026 Sep 21;20(9):e0014203. doi: 10.1371/journal.pntd.0014203 (PMC13641699; doi:10.1371/journal.pntd.0014203)
Supplement: Supplementary file 4 [file pntd.0014203.s007.docx]

**Supplementary Table 2.** Population-structure model evaluation using fastSTRUCTURE and ADMIXTURE. Marginal likelihood, cross-validation values, and standard deviations are shown for K = 1–12 in *Sporothrix brasiliensis* and *Sporothrix schenckii*. The model components supported K = 6 for *S. brasiliensis* and K = 2 for *S. schenckii*, consistent with the major phylogenetic groups recovered in the SNP-based analyses.

|  | *S. brasiliensis* | | | *S. schenckii* | | |
| --- | --- | --- | --- | --- | --- | --- |
| **K value** | **Marginal Likelihood** | **Cross**  **Validation** | **Standard Deviation** | **Marginal Likelihood** | **Cross**  **Validation** | **Standard Deviation** |
| 1 | -0.627 | 0.629 | 0.025 | -1.086 | 1.147 | 0.025 |
| 2 | -0.274 | 0.175 | 0.022 | -0.814 | 0.757 | 0.024 |
| 3 | -0.310 | 0.240 | 0.011 | -0.814 | 0.739 | 0.046 |
| 4 | -0.274 | 0.175 | 0.022 | -0.814 | 0.744 | 0.035 |
| 5 | -0.254 | 0.142 | 0.012 | -0.814 | 0.746 | 0.022 |
| 6 | -0.254 | 0.153 | 0.021 | -0.814 | 0.740 | 0.019 |
| 7 | -0.254 | 0.155 | 0.020 | -0.814 | 0.765 | 0.051 |
| 8 | -0.247 | 0.132 | 0.010 | -0.814 | 0.751 | 0.029 |
| 9 | -0.247 | 0.140 | 0.009 | -0.814 | 0.747 | 0.018 |
| 10 | -0.247 | 0.135 | 0.014 | -0.814 | 0.745 | 0.037 |
| 11 | -0.247 | 0.136 | 0.009 | -0.814 | 0.743 | 0.032 |
| 12 | -0.247 | 0.127 | 0.013 | -0.843 | 0.791 | 0.029 |

* Lowest K value at the marginal-likelihood plateau: *S. brasiliensis*, K = 8; *S. schenckii*, K = 2.

** Model components used to explain structure in the data: *S. brasiliensis*, K = 6; *S. schenckii*, K = 2.
