# Supplementary material for: Genome variation of Sporothrix schenckii and Sporothrix brasiliensis
Source: PLoS Negl Trop Dis. 2026 Sep 21;20(9):e0014203. doi: 10.1371/journal.pntd.0014203 (PMC13641699; doi:10.1371/journal.pntd.0014203)
Supplement: Supplementary file 5 [file pntd.0014203.s013.docx]

**Response to Reviewers' Comments:**

**Methods**

Reviewer #1: See below

Reviewer #2: The methodology used for annotation of genes identified in the GWAS analysis (e.g. Gene Ontology analyses) is first introduced in the Results section (Line 377). This information should instead be described in the Methods section.

A: We thank the reviewer for this helpful comment. We agree that the annotation of GWAS-associated variants and the Gene Ontology analyses should be described in the Methods. We have revised the Methods section to clarify that significant GWAS-associated variant sites were annotated by parsing the GFF3 annotation file of the S. brasiliensis reference genome RefSeq GCF_000820605.1. We also added details on how variants were classified relative to predicted coding features and neighboring annotated regions. Finally, we clarified that GO enrichment results were summarized and visualized using REVIGO. See lines 280-295

Reviewer #3: Overall, this section is well presented, although there are some points that should be carefully reviewed, as outlined below.

1. Line 64 (introduction): The term “species complex” should no longer be used to describe the closely related species listed by the authors, even if they were initially reported as cryptic species within the S. schenckii population. According to Chen et al. (2016, doi: 10.1016/j.funbio.2015.09.003), a species complex is defined as a group of closely related organisms that are difficult to distinguish. The “S. schenckii group” comprises only four species, which are well distinguishable and therefore do not constitute a complex; instead, they form the so-called “pathogenic clade,” as reported by de Beer et al. (2016), which the authors cited in the paper (see Reference 3).

A: We have revised the text and replaced “S. schenckii species complex” with “pathogenic clade,” which more accurately reflects the evolutionary relationship among these clinically relevant species. See lines 63-66

2. Line 112: How many S. brasiliensis and how many S. schenckii isolates were specifically sequenced? Please report these numbers clearly in the main text, rather than only in the supplementary data.

A: We thank the reviewer for this comment. We have now clarified this information in the main text. Specifically, we sequenced 13 new Sporothrix isolates, comprising 11 S. brasiliensis and 2 S. schenckii isolates. See line 120

3. Line 150: The Fisher Strand Bias cutoff set to “≥ 60.0” appears unusual; is this perhaps a typo, as such a threshold would typically retain highly biased calls?
Additionally, why were SNPs located in repetitive regions not filtered out? This is particularly important for downstream phylogenetic analyses, which assume relatedness among individuals based on independent mutation events; including such sites may violate the assumptions of phylogenetic inference. Furthermore, no PCR duplicate removal step is described in the pipeline, which may negatively affect variant call reliability. Please provide the full analysis scripts, including all commands used, or re-run the analysis with corrected parameters.

A: We thank the reviewer for identifying this issue. The Fisher Strand Bias threshold was incorrectly described in the Methods due to a typographical error. Variants with FS ≥ 60.0 were not retained; instead, retained SNPs were required to have FS ≤ 60.0, consistent with the intended GATK hard-filtering strategy. We have revised the Methods to clarify the filtering criteria, including the additional exclusion of SNPs with coverage <10×, variant allele frequency <90%, and SNPs located within putative duplicated regions identified by NUCmer. We have also made the full analysis scripts and command lines available at <https://gitlab.com/ujwalbagal/sporothrix-project/-/tree/main/For_manuscript?ref_type=heads>.

4. Lines 162–165: Please expand on this methodological section. The statement “Phylogenies were inferred from whole-genome single nucleotide polymorphisms (SNPs) as well as from individual scaffolds derived from S. brasiliensis reference genomes to evaluate genealogical concordance among genome segments” requires clarification. Which reference genomes were used? In lines 142-143, S. brasiliensis (GCF_000820605.1) and S. schenckii (GCA_000961545.1) reference genomes are reported. How were SNPs called against different reference coordinate systems integrated into a single VCF file? Were the genomes of the other strains assembled de novo and aligned to reference? If so, this analysis should be explicitly described in Materials and Methods.

A: We thank the reviewer for highlighting the need for clarification. We have revised the Materials and Methods to explicitly describe the reference-based mapping strategy. For SNP-based phylogenomic analyses, all FASTQ reads were aligned to the S. brasiliensis reference genome GCF_000820605.1, ensuring that all variants were called within a single reference coordinate system and integrated into one unified VCF file. For CNV analyses, reads were mapped in a species-specific manner: S. brasiliensis isolates were aligned to the S. brasiliensis reference genome GCF_000820605.1, whereas S. schenckii isolates were aligned to the S. schenckii reference genome GCA_000961545.1. We have also revised the text to clarify that no SNP datasets generated from different reference coordinate systems were merged.

5. Lines 162–165: Were S. schenckii reads aligned to the S. brasiliensis reference genome? If so, this would likely underestimate the number of variants in S. schenckii, as a substantial fraction of reads may fail to align or may align incorrectly. Moreover, if a single multi-sample VCF containing both S. brasiliensis and S. schenckii was generated and used for population structure analyses, this could compromise the robustness of the results, especially given the limited sample size for S. schenckii. If this is not the case, please provide the full analysis pipeline, preferably via a public repository (e.g., GitHub) or in supplementary materials, to ensure reproducibility and transparency.

**A:** We thank the reviewer for this important point. For SNP-based phylogenomic reconstruction, reads from both *S. brasiliensis* and *S. schenckii* were mapped to the *S. brasiliensis* reference genome GCF_000820605.1 to generate a unified genome-wide SNP matrix within a single reference coordinate system. We agree that this approach may underestimate the total number of variants in *S. schenckii* because of interspecific divergence and reduced or biased read mapping. Accordingly, the combined-species SNP dataset was used only for broad phylogenomic placement and not for fine-scale population-genetic inference. For population genetic and CNV analyses, we used species-specific mapping and SNP-calling workflows. *S. brasiliensis* reads were mapped to the *S. brasiliensis* reference genome, while *S. schenckii* reads were mapped to the *S. schenckii* reference genome GCA_000961545.1. The resulting species-specific BAM and VCF files were used for PCA, fastStructure, and CNV analyses within each species. We have revised the Methods section to clarify this distinction and will provide the full pipeline in a public repository to ensure reproducibility.

6. The authors should provide the raw sequencing reads to the reviewers. This can be done via a private link for reviewers, even if the data are not intended for immediate public release.

A: The raw sequencing reads have been deposited in the NCBI Sequence Read Archive (SRA) and are available under accession numbers: SRR39070006, SRR39070009, SRR39070008, SRR39070007, SRR39070005, SRR39070004, SRR39070003, SRR39070002, SRR39070001, SRR39070000, SRR39069999, SRR39069998 and SRR39069997. These accession numbers have now been included in the Data Availability section and Supplementary Table 1 of the revised manuscript to ensure that reviewers and readers can access the sequencing data.

Figures 1A and 2.

Figure 1A: What criteria were used to define clusters in the phylogenetic tree? Please include bootstrap support values or provide a corresponding legend. How was the reference genome incorporated into the phylogeny, and how was a SNP-based tree generated across both species?
Additionally, the SNP counts previously reported (610,242 and 1,474,627; see lines 235–237) do not sum to 2,274,664 as stated in the Figure 1 legend (“Maximum-likelihood (ML) phylogeny inferred from 2,274,664 genome-wide SNPs showing a strong separation between S. brasiliensis and S. schenckii isolates”). Please clarify the origin of this number.

A: We thank the reviewer for these important observations. We have revised the Figure 1 legend M&M and the Results sections to clarify how phylogenetic clusters were defined and how the SNP datasets were generated. Clusters in the phylogenetic tree were defined as monophyletic groups recovered in the whole-genome SNP phylogeny and further evaluated for genealogical concordance across individual scaffolds. We also considered branch support based on UFboot, aBayes, and SH-aLRT values, which have now been added to the tree. In addition, we assessed whether the same clusters were recovered as genetic populations under the variational Bayesian framework implemented in fastSTRUCTURE. Regarding SNP calling, the SNP-based phylogeny shown in Fig. 1A was generated from genome-wide SNPs called using a shared reference-based framework, as clarified in the revised Methods and in our responses above. The reference genome was included as the coordinate framework for SNP calling and, when appropriate, as a reference tip in the phylogeny. For population genetic analyses, however, we used species-restricted SNP datasets to avoid biases associated with interspecific divergence, differential read mapping, shared ancestral polymorphisms, or sites potentially affected by incomplete lineage sorting. Specifically, population genetic analyses restricted to *S. brasiliensis* were performed using 610,242 species-specific SNPs, whereas analyses restricted to *S. schenckii* were performed using 1,474,627 species-specific SNPs. These analyses yielded concordant results across the maximum-likelihood phylogeny, PCA, and fastSTRUCTURE analyses. We also agree that the SNP counts required clarification. The 610,242 and 1,474,627 SNPs correspond to species-restricted SNP datasets used for within-species population genetic analyses and were not intended to sum to the 2,274,664 genome-wide SNPs used for the broader phylogenomic tree.

Figure 2: Please avoid referring to contigs as chromosomes. This should be corrected in this figure and consistently throughout the manuscript.

A: We agree that these genomic units should not be referred to as chromosomes, as the current reference assemblies are scaffold/contig-level assemblies rather than chromosome-level assemblies. We have revised Figure 2 and the corresponding legend to replace chromosome labels with scaffold numbers. We also checked the manuscript and corrected this terminology consistently throughout the text, figures, and figure legends.

**Conclusions**


-Are the conclusions supported by the data presented?

-Are the limitations of analysis clearly described?

-Do the authors discuss how these data can be helpful to advance our understanding of the topic under study?

-Is public health relevance addressed?

Reviewer #1: See below

Reviewer #2: Although several caveats are addressed in the discussion, it would be valuable to also acknowledge a limitation related to the CNV analyses of S. schenckii. The level of phylogenetic divergence between the two clades may hinder accurate CNV detection using a single reference genome, which could have impacted the results.

A: We agree that CNV inference in Sporothrix may be affected by the phylogenetic divergence between clades, particularly because depth-based CNV detection relies on efficient and unbiased read mapping to a reference genome. Differences in sequence divergence, local genome structure, and mapping efficiency may therefore influence CNV estimates when a single reference is used. We have added this point as a limitation in the Discussion section.

Reviewer #3: This part of the manuscript is overall well presented, and the conclusions appear to be supported by the data provided.

**Editorial and Data Presentation Modifications?**

Use this section for editorial suggestions as well as relatively minor modifications of existing data that would enhance clarity. If the only modifications needed are minor and/or editorial, you may wish to recommend “Minor Revision” or “Accept”.

Reviewer #1: See below

Reviewer #2: Please consider the following minor revisions, which will strengthen the manuscript:

Editorial
Lines 130/133/134/144 and 236 – Please ensure the number of isolates reported is accurate in each case. There seems to be a discrepancy between the text and Table S1.

A: We have corrected the number of isolates reported in the text and ensured consistency across the manuscript and Table S1.,

Line 194 – Clarify that the assemblies being referred to are of the reference genomes.

A: We thank the reviewer for this comment. We have clarified this point in the revised manuscript, including the relevant sections of the Materials and Methods and the Results sections

Line 208 – “, a read depth-based approach, applied to the realigned BAM files [42].” changed to “,a tool that uses a read-depth based approach, was applied to the realigned BAM files [42].”

A: We have corrected this as follows: To detect CNVs associated with genomic alterations such as gains and losses, Control-FREEC, a tool that uses a read-depth-based approach, was applied to the realigned BAM files [42].

Line 219 – Add a space after full stop.

A: space added.

Lines 283-284 – Please maintain consistency with clade names. (Clade C1 / Clade C-1)

A: This was corrected along the manuscript including the Figure 1 and Table1

Line 285 – “primarily” suggests most, however all strains considered were of MAT1-1. Revise sentence to better portray this.

A: We have modified this sentence as follows: It is worth nothing that Sb Clade C-I contains only isolates with the MAT1-1 idiomorph in the present dataset, whereas Sb Clade C-II is populated by the MAT1-2 allele, suggesting two independent clonal expansions that occurred between 2017 and 2021 within São Paulo state of Brazil.

Line 323 – Remove “with” for clarity

A: Word removed

Lines 326, 372, 376 – For consistency, ensure reference to figures and tables in the text, e.g. (Fig. 2 B-C), is in bold.

A: Thanks for capturing this. The fiugure numbering/style was modified in the entire manuscript

Line 401 – Change “(Table I)” to “(Table 1)”

A: This was modified

Figure legends
Line 680 and 682 – Bold “Sporothrix” and “Sporothrix brasiliensis”

A: This was modified

Line 714 – “S. brasiliensis” must be changed to “S. schenckii”

A: This was modified

Supplementary tables 2 and 3 – Tables are incorrectly labelled as in the supplementary files, please correct.

A: This was modified

In-text references
Line 69 – Provide reference for soil/ plant association

A: Reference added

Line75/83 – Provide reference for differences in virulence

A: Reference included

Line 88 – Provide reference for “most closely related species is S. globosa”

A: Reference added

Line 93 – Provide reference for “variation of pigmentation and growth rate”

A: Reference added

Line 156 – Instead of “(see[10])”, consider “[10]”

A: This was modified accordingly

Lines 216/217 – Provide references for statistical tests

A:We thank the reviewer for this comment. We have clarified in the revised Materials and Methods that CNVs were inferred using Control-FREEC, whereas downstream statistical comparisons of CNV content among lineages were performed in R using non-parametric tests implemented in the stats package, including wilcox.test and ks.test.

Lines 225/226 – Cite article that reported MICs

A: Reference was added to the manuscript

Reference list

Please add relevant issue/ page number information for reference:

1
Please correct title capitalisation in the following references: 6,7,19,20,32,41,44,46,51,52,53,55,56
Please provide abbreviated journal name for the following:
3,49,54
The inclusion of “Epud” dates in some of the reference list is inconsistent. Please remove these from the references.

A: These were modified in the entire reference list

Reviewer #3: Accept

**Summary and General Comments**


Use this section to provide overall comments, discuss strengths/weaknesses of the study, novelty, significance, general execution and scholarship. You may also include additional comments for the author, including concerns about dual publication, research ethics, or publication ethics. If requesting major revision, please articulate the new experiments that are needed.

Reviewer #1: The manuscript analyzes a relevant subject, the genetic variations of Sporothrix species. In a strict sense, this is not the first approach to address this subject. However, the plus side of this study is the use of whole-genome information to reach the main conclusions. Moreover, the study, unlike others, includes a significant amount of analyzed genomes.

The main shortcomings are:

The lack of an inclusive approach to gather strains from around the world. The sequences are basically from America (South America), and the strains may not have the same genomic features as isolates from Asia or Africa, for example.
A: We thank the reviewer for this important point. We agree that larger and more geographically balanced sampling would improve the resolution of the analyses. However, well-characterized Sporothrix isolates are not widely available, particularly from underrepresented regions and host sources. Our aim was therefore to establish a reproducible genomic strategy to address this limitation, which can be replicated and expanded as new isolates become available. We have now clarified this point in the revised manuscript.

Even though the authors focused the analysis on relevant genomic features, the analysis is limited in terms of all the information that may be extracted from a genomic analysis. The gain and loss of putative genes are described and analyzed in a superficial manner. Are there particular loss/gain features in clades? Are there expansions/constrictions in gene families that allow the authors to propose hypotheses on the species-specific traits? A thorough analysis in this an other subjects should be incorporated. The search and distribution of transposable elements is absent, and this has been documented to be a key molecular mechanism in species evolution.
A: We thank the reviewer for this thoughtful suggestion. We agree that specific gene gain/loss events associated with population-specific CNV patterns are biologically relevant. Although we observed such patterns and reported them in the manuscript, we did not explore them in depth because they require validation with additional strains, improved genome assemblies, and functional assays. TE analyses were performed and incorporated into the CNV plots to provide genomic context. We have clarified these limitations in the revised Discussion and now indicate that ongoing work using telomere-to-telomere long-read assemblies will allow a more detailed investigation of CNVs, transposable elements, and lineage-specific gene gain/loss events.

The association between SNP and itraconazole resistance required additional evidence to support the claims included in the manuscript. Experimental evidence showing that a particular change in an SNP renders an itraconazole-sensitive strain into an itraconazole-resistant one should be included as part of the study. How can these SNPs account for the phenotypic change if they are not part of genes? If they belong to a gene, how does the sequence change affect genetic function?

A: We thank the reviewer for this important comment. We agree that these aspects represent relevant limitations of the current study and should be interpreted with caution. As noted above, population-specific CNV patterns and putative gene gain/loss events were observed, but their biological significance requires further validation using broader sampling, improved genome assemblies, and functional assays. We have clarified these points in the revised manuscript and expanded the Discussion to explicitly acknowledge these limitations.

I know that this is a subject of debate between bioinformaticians and researchers based on wet laboratories. Genomic analysis does not allow gene identification. The methodology is robust enough to predict ORFs, which are only a part of a gene. In other words, a gene is not equal to ORF. Moreover, the identification of an ORF does not obligate a sequence to be part of a gene; please keep in mind the presence of pseudogenes. For the sake of research integrity, the auhtors should refer to sequences as predicted ORFS, nor genes. In a similar sense, locus/loci are positions for genes, not for ORFs; the authors should refrain from using these terms.
A: We agree that, in the context of genome annotation and comparative genomics, the term “gene” should be used cautiously when referring to computationally predicted coding features. We have revised the manuscript to avoid overstating annotation certainty and have replaced “genes” with “predicted ORFs,” “predicted coding sequences,” or “annotated coding features” where appropriate. Similarly, we revised the use of “locus/loci” in the GWAS section to refer more precisely to “genomic positions,” “variant sites,” or “annotated regions.”

In the same sense, bioinformatics approach predict functions of putative genes, but whehter there is indeed a transcriptional process on the sequence and whether the RNA undergoes translation are facts not possible to be predicted from the genomic analysis. So, it is wrong to talk about genes encoding X or Y proteins with particular functions. The authors should change the tone to putative ORFs encoding putative proteins with X or Y function.

A: This was modifies accordingly as discussed before.

I did not find the codes of the sequences deposited in bioinformatic repositories.

A: We have rechecked all accession numbers listed in the manuscript and supplementary tables directly in the NCBI database, and all records were confirmed to be available/searchable.

Reviewer #2: The study "Genome variation of Sporothrix schenckii and Sporothrix brasiliensis" (PNTD-D-26-00623), provides novel insights into the genomic variation of two major Sporothrix pathogens, S. brasiliensis and S. schenckii, highlighting differences in these species that have important epidemiological and evolutionary implications. It is an original and relevant work, that I believe will interest readers of PLOS Neglected Tropical Diseases. However, the suggested revisions, including minor clarification of methodology and limitations, as well as improvements to grammar and referencing, would help strengthen the manuscript further. With these minor revisions, I believe this study will make a valuable contribution to sporotrichosis research. In terms of data accessibility, accession numbers have been included for all the previously sequenced isolates. However, accession numbers for the sequence data generated in this paper should be updated as they are made available.

A: We thank the reviewer for this request. All raw sequencing reads generated in this study have been uploaded to the NCBI Sequence Read Archive (SRA) under the following deposition numbers:

| **accession** | **study** | **object_status** | **bioproject_accession** | **biosample_accession** | **sample_name** | **library_ID** |
| --- | --- | --- | --- | --- | --- | --- |
| **SRR39070009** | SRP278217 | new | PRJNA607682 | SAMN60719601 | Sb19536 | SB001 |
| **SRR39070008** | SRP278217 | new | PRJNA607682 | SAMN60719602 | M4559 | SB002 |
| **SRR39070004** | SRP278217 | new | PRJNA607682 | SAMN60719603 | Sb19541 | SB003 |
| **SRR39070003** | SRP278217 | new | PRJNA607682 | SAMN60719604 | 98-5 | SB004 |
| **SRR39070002** | SRP278217 | new | PRJNA607682 | SAMN60719605 | 17878 | SB005 |
| **SRR39070001** | SRP278217 | new | PRJNA607682 | SAMN60719606 | Gato 1 | SB006 |
| **SRR39070000** | SRP278217 | new | PRJNA607682 | SAMN60719607 | GATO 3 | SB007 |
| **SRR39069999** | SRP278217 | new | PRJNA607682 | SAMN60719608 | M1078 | SB008 |
| **SRR39069998** | SRP278217 | new | PRJNA607682 | SAMN60719609 | M1878 | SB009 |
| **SRR39069997** | SRP278217 | new | PRJNA607682 | SAMN60719610 | M3952 | SB0010 |
| **SRR39070007** | SRP278217 | new | PRJNA607682 | SAMN60719611 | B19089 | SB0011 |
| **SRR39070006** | SRP278217 | new | PRJNA607682 | SAMN60719612 | B19090 | SS001 |
| **SRR39070005** | SRP278217 | new | PRJNA607682 | SAMN60719613 | B19092 | SS002 |

Reviewer #3: The manuscript is generally well written and well organized. However, it requires some careful revisions in the Materials and Methods section (see my specific comments).

A: Modifications were done accordingly
