# Supplementary material for: Genome variation of Sporothrix schenckii and Sporothrix brasiliensis
Source: PLoS Negl Trop Dis. 2026 Sep 21;20(9):e0014203. doi: 10.1371/journal.pntd.0014203 (PMC13641699; doi:10.1371/journal.pntd.0014203)
Supplement: Supplementary file 6 [file pntd.0014203.s014.docx]

**Response to Reviewers' Comments:**

Reviewer's Responses to Questions

**Key Review Criteria Required for Acceptance?**


As you describe the new analyses required for acceptance, please consider the following:


**Methods**


-Are the objectives of the study clearly articulated with a clear testable hypothesis stated?

-Is the study design appropriate to address the stated objectives?

-Is the population clearly described and appropriate for the hypothesis being tested?

-Is the sample size sufficient to ensure adequate power to address the hypothesis being tested?

-Were correct statistical analysis used to support conclusions?

-Are there concerns about ethical or regulatory requirements being met?

Reviewer #1: See below

Reviewer #2: (No Response)

Reviewer #3: The authors have addressed all the technical issues raised during the first round of peer review.

**A: We thank the reviewers for their careful assessment of the revised manuscript. We are pleased that the technical issues raised during the first round of peer review have been addressed.**

**Results**


-Does the analysis presented match the analysis plan?

-Are the results clearly and completely presented?

-Are the figures (Tables, Images) of sufficient quality for clarity?

Reviewer #1: See below

Reviewer #2: (No Response)

Reviewer #3: The authors have addressed all the issues raised during the first round of peer review.

**A: We thank the reviewers for their evaluation. We are pleased that the Results section now adequately addresses the issues raised during the first round of peer review.**

**Conclusions**


-Are the conclusions supported by the data presented?

-Are the limitations of analysis clearly described?

-Do the authors discuss how these data can be helpful to advance our understanding of the topic under study?

-Is public health relevance addressed?

Reviewer #1: See below

Reviewer #2: (No Response)

Reviewer #3: Overall, this section is clearly presented and the conclusions are adequately supported by the data.

**A: We thank the reviewer for this positive assessment.**

**Editorial and Data Presentation Modifications?**


Use this section for editorial suggestions as well as relatively minor modifications of existing data that would enhance clarity. If the only modifications needed are minor and/or editorial, you may wish to recommend “Minor Revision” or “Accept”.

Reviewer #1: (No Response)

Reviewer #2: (No Response)

Reviewer #3: Accept

**Summary and General Comments**


Use this section to provide overall comments, discuss strengths/weaknesses of the study, novelty, significance, general execution and scholarship. You may also include additional comments for the author, including concerns about dual publication, research ethics, or publication ethics. If requesting major revision, please articulate the new experiments that are needed.

Reviewer #1: I thank the authors for the improvements to the manuscript and for their comments on my observations. As the authors mentioned in the rebuttal letter, there is a lot to do to refine the analysis, and currently, they are sitting on a bunch of interesting data that are waiting for a thorough and robust analysis. As I mentioned in my first assessment, the analysis is superficial and needs more work. The weakest part of the study is related to the putative SNPs linked to antifungal resistance; without additional experimental validation, the authors do not have sufficient evidence to sustain the drawn conclusions. My recommendation is to halt the publication of the study as it is, to invest time to complement it, and then to seek the publication. Such a version would be a strong contribution to the field.

Response:

We thank the reviewer for this thoughtful comment and fully agree that experimental validation will ultimately be required to establish causal relationships between the candidate variants identified here and antifungal resistance. However, we respectfully note that the primary objective of this study was not to functionally validate resistance determinants, but rather to establish the first genome-wide population genomic framework for pathogenic *Sporothrix* spp. and to identify candidate loci associated with antifungal susceptibility. Genome-wide association studies (GWAS) are inherently hypothesis-generating approaches that identify statistically supported candidate loci for subsequent functional investigation rather than demonstrating causality. This strategy has been widely adopted in fungal genomics and has substantially accelerated the discovery of genes involved in antifungal resistance, virulence, and host adaptation across numerous pathogenic fungi. To our knowledge, this represents the first GWAS performed in *Sporothrix* and one of the first comprehensive population genomic analyses integrating phylogenomics, copy number variation, and association mapping in an endemic dimorphic fungal pathogen. Beyond the biological findings themselves, this work establishes a reproducible analytical framework that can now be applied to larger *Sporothrix* collections and extended to other emerging fungal pathogens. Following the reviewer's suggestion, we have strengthened the Results and Discussion sections to more explicitly acknowledge that the loci identified should be considered candidate variants whose functional roles remain to be experimentally validated. We believe this clarification appropriately reflects both the strengths and the limitations of the present study while maintaining conclusions that are fully supported by the genomic data.

Reviewer #2: The article "Genome variation of Sporothrix schenckii and Sporothrix brasiliensis" is an interesting and informative study that expands current knowledge on two major Sporothrix pathogens. Using genomic approaches the authors explored the genetic diversity of the two Sporothrix pathogens, and related these insights back to epidemiological data, providing valuable insights into their population structure, reproduction and dissemination. This work is timely considering the current state of sporotrichosis and the growing relevance of feline driven outbreaks in both pathogens. Furthermore, the genomic data generated in this study provide valuable resources for future genetic diversity and comparative genomic studies. The authors adequately considered all the suggestions from the last round of review and I believe the article is suitable for publication in the current form.

A: We thank the reviewer for the positive assessment and for considering the manuscript suitable for publication in its current form.

Reviewer #3: (No Response)

PLOS authors have the option to publish the peer review history of their article ([what does this mean?](https://track.editorialmanager.com/CL0/https:%2F%2Fjournals.plos.org%2Fplosntds%2Fs%2Feditorial-and-peer-review-process%23loc-peer-review-history/1/010f019f211d18fe-52e0cdae-9586-4356-99c3-b9cb66a74351-000000/yp6Gbad1W62r11L7huLp3fVpaYJtjQIGJUq9huOt-rc=258)). If published, this will include your full peer review and any attached files.


If you choose “no”, your identity will remain anonymous but your review may still be made public.


**Do you want your identity to be public for this peer review?** For information about this choice, including consent withdrawal, please see our [Privacy Policy](https://track.editorialmanager.com/CL0/https:%2F%2Fwww.plos.org%2Fprivacy-policy/1/010f019f211d18fe-52e0cdae-9586-4356-99c3-b9cb66a74351-000000/RObST_MdoYcjTU4ii2LnLZLLWPF6NK8CVsPfOJsAx5k=258).

Reviewer #1: No

Reviewer #2: No

Reviewer #3: No
