# Supplementary material for: Genome variation of Sporothrix schenckii and Sporothrix brasiliensis
Source: PLoS Negl Trop Dis. 2026 Sep 21;20(9):e0014203. doi: 10.1371/journal.pntd.0014203 (PMC13641699; doi:10.1371/journal.pntd.0014203)
Supplement: Peer review history

# Peer review: Decision Letter 0

**DOI:** 10.1371/journal.pntd.0014203.r001

**Authors:** Marcio Rodrigues

23 Apr 2026

Genome variation of Sporothrix schenckii and Sporothrix brasiliensis

PLOS Neglected Tropical Diseases

Dear Dr. Teixeira,

Thank you for submitting your manuscript to PLOS Neglected Tropical Diseases. After careful consideration, we feel that it has merit but does not fully meet PLOS Neglected Tropical Diseases's publication criteria as it currently stands. Therefore, we invite you to submit a revised version of the manuscript that addresses the points raised during the review process.

Please submit your revised manuscript within by Jun 22 2026 11:59PM. If you will need more time than this to complete your revisions, please reply to this message or contact the journal office at plosntds@plos.org. When you're ready to submit your revision, log on to https://www.editorialmanager.com/pntd/ and select the 'Submissions Needing Revision' folder to locate your manuscript file.

Please include the following items when submitting your revised manuscript:

* A letter that responds to each point raised by the editor and reviewer(s). You should upload this letter as a separate file labeled 'Response to Reviewers'. This file does not need to include responses to any formatting updates and technical items listed in the 'Journal Requirements' section below.

* A marked-up copy of your manuscript that highlights changes made to the original version. You should upload this as a separate file labeled 'Revised Manuscript with Track Changes'.

* An unmarked version of your revised paper without tracked changes. You should upload this as a separate file labeled 'Manuscript'.

If you would like to make changes to your financial disclosure, competing interests statement, or data availability statement, please make these updates within the submission form at the time of resubmission. Guidelines for resubmitting your figure files are available below the reviewer comments at the end of this letter.

We look forward to receiving your revised manuscript.

Kind regards,

Marcio L Rodrigues

Section Editor

PLOS Neglected Tropical Diseases

Marcio Rodrigues

Section Editor

PLOS Neglected Tropical Diseases

Shaden Kamhawi

co-Editor-in-Chief

PLOS Neglected Tropical Diseases

orcid.org/0000-0003-4304-636XX

Paul Brindley

co-Editor-in-Chief

PLOS Neglected Tropical Diseases

orcid.org/0000-0003-1765-0002

**Journal Requirements:**

1) <carina-action-element class="ng-star-inserted">Please ensure that the CRediT author contributions listed for every co-author are completed accurately and in full.</carina-action-element>

2) <carina-action-element class="ng-star-inserted">Please provide an Author Summary. This should appear in your manuscript between the Abstract (if applicable) and the Introduction, and should be 150-200 words long. The aim should be to make your findings accessible to a wide audience that includes both scientists and non-scientists. Sample summaries can be found on our website under Submission Guidelines:</carina-action-element>

3) <carina-action-element class="ng-star-inserted">We do not publish any copyright or trademark symbols that usually accompany proprietary names, eg ©, ®, or TM (e.g. next to drug or reagent names). Therefore please remove all instances of trademark/copyright symbols throughout the text, including:</carina-action-element>

4) <carina-action-element class="ng-star-inserted">Please upload all main figures as separate Figure files in .tif or .eps format. For more information about how to convert and format your figure files please see our guidelines: </carina-action-element>

5)

**Reviewers' Comments:**

Reviewer's Responses to Questions

**Key Review Criteria Required for Acceptance?**

As you describe the new analyses required for acceptance, please consider the following:

**Methods**

-Are the objectives of the study clearly articulated with a clear testable hypothesis stated?

-Is the study design appropriate to address the stated objectives?

-Is the population clearly described and appropriate for the hypothesis being tested?

-Is the sample size sufficient to ensure adequate power to address the hypothesis being tested?

-Were correct statistical analysis used to support conclusions?

-Are there concerns about ethical or regulatory requirements being met?

Reviewer #1: See below

Reviewer #2: The methodology used for annotation of genes identified in the GWAS analysis (e.g. Gene Ontology analyses) is first introduced in the Results section (Line 377). This information should instead be described in the Methods section.

Reviewer #3: Overall, this section is well presented, although there are some points that should be carefully reviewed, as outlined below.

1. Line 64 (introduction): The term “species complex” should no longer be used to describe the closely related species listed by the authors, even if they were initially reported as cryptic species within the S. schenckii population. According to Chen et al. (2016, doi: 10.1016/j.funbio.2015.09.003), a species complex is defined as a group of closely related organisms that are difficult to distinguish. The “S. schenckii group” comprises only four species, which are well distinguishable and therefore do not constitute a complex; instead, they form the so-called “pathogenic clade,” as reported by de Beer et al. (2016), which the authors cited in the paper (see Reference 3).

2. Line 112: How many S. brasiliensis and how many S. schenckii isolates were specifically sequenced? Please report these numbers clearly in the main text, rather than only in the supplementary data.

3. Line 150: The Fisher Strand Bias cutoff set to “≥ 60.0” appears unusual; is this perhaps a typo, as such a threshold would typically retain highly biased calls?

Additionally, why were SNPs located in repetitive regions not filtered out? This is particularly important for downstream phylogenetic analyses, which assume relatedness among individuals based on independent mutation events; including such sites may violate the assumptions of phylogenetic inference. Furthermore, no PCR duplicate removal step is described in the pipeline, which may negatively affect variant call reliability. Please provide the full analysis scripts, including all commands used, or re-run the analysis with corrected parameters.

4. Lines 162–165: Please expand on this methodological section. The statement “Phylogenies were inferred from whole-genome single nucleotide polymorphisms (SNPs) as well as from individual scaffolds derived from S. brasiliensis reference genomes to evaluate genealogical concordance among genome segments” requires clarification. Which reference genomes were used? In lines 142-143, S. brasiliensis (GCF_000820605.1) and S. schenckii (GCA_000961545.1) reference genomes are reported. How were SNPs called against different reference coordinate systems integrated into a single VCF file? Were the genomes of the other strains assembled de novo and aligned to reference? If so, this analysis should be explicitly described in Materials and Methods.

5. Lines 162–165: Were S. schenckii reads aligned to the S. brasiliensis reference genome? If so, this would likely underestimate the number of variants in S. schenckii, as a substantial fraction of reads may fail to align or may align incorrectly. Moreover, if a single multi-sample VCF containing both S. brasiliensis and S. schenckii was generated and used for population structure analyses, this could compromise the robustness of the results, especially given the limited sample size for S. schenckii. If this is not the case, please provide the full analysis pipeline, preferably via a public repository (e.g., GitHub) or in supplementary materials, to ensure reproducibility and transparency.

6. The authors should provide the raw sequencing reads to the reviewers. This can be done via a private link for reviewers, even if the data are not intended for immediate public release.

**********

**Results**

-Does the analysis presented match the analysis plan?

-Are the results clearly and completely presented?

-Are the figures (Tables, Images) of sufficient quality for clarity?

Reviewer #1: See below

Reviewer #2: (No Response)

Reviewer #3: Overall, this section is well organized. I have a few specific comments regarding Figures 1A and 2.

Figure 1A: What criteria were used to define clusters in the phylogenetic tree? Please include bootstrap support values or provide a corresponding legend. How was the reference genome incorporated into the phylogeny, and how was a SNP-based tree generated across both species?

Additionally, the SNP counts previously reported (610,242 and 1,474,627; see lines 235–237) do not sum to 2,274,664 as stated in the Figure 1 legend (“Maximum-likelihood (ML) phylogeny inferred from 2,274,664 genome-wide SNPs showing a strong separation between S. brasiliensis and S. schenckii isolates”). Please clarify the origin of this number.

Figure 2: Please avoid referring to contigs as chromosomes. This should be corrected in this figure and consistently throughout the manuscript.

**********

**Conclusions**

-Are the conclusions supported by the data presented?

-Are the limitations of analysis clearly described?

-Do the authors discuss how these data can be helpful to advance our understanding of the topic under study?

-Is public health relevance addressed?

Reviewer #1: See below

Reviewer #2: Although several caveats are addressed in the discussion, it would be valuable to also acknowledge a limitation related to the CNV analyses of S. schenckii. The level of phylogenetic divergence between the two clades may hinder accurate CNV detection using a single reference genome, which could have impacted the results.

Reviewer #3: This part of the manuscript is overall well presented, and the conclusions appear to be supported by the data provided.

**********

**Editorial and Data Presentation Modifications?**

Use this section for editorial suggestions as well as relatively minor modifications of existing data that would enhance clarity. If the only modifications needed are minor and/or editorial, you may wish to recommend “Minor Revision” or “Accept”.

Reviewer #1: See below

Reviewer #2: Please consider the following minor revisions, which will strengthen the manuscript:

Editorial

Lines 130/133/134/144 and 236– Please ensure the number of isolates reported is accurate in each case. There seems to be a discrepancy between the text and Table S1.

Line 194 – Clarify that the assemblies being referred to are of the reference genomes.

Line 208 – “, a read depth-based approach, applied to the realigned BAM files [42].” changed to “,a tool that uses a read-depth based approach, was applied to the realigned BAM files [42].”

Line 219 – Add a space after full stop.

Lines 283-284 – Please maintain consistency with clade names. (Clade C1 / Clade C-1)

Line 285 – “primarily” suggests most, however all strains considered were of MAT1-1. Revise sentence to better portray this.

Line 323 – Remove “with” for clarity

Lines 326, 372, 376 – For consistency, ensure reference to figures and tables in the text, e.g. (Fig. 2 B-C), is in bold.

Line 401 – Change “(Table I)” to “(Table 1)”

Figure legends

Line 680 and 682 – Bold “Sporothrix” and “Sporothrix brasiliensis”

Line 714 – “S. brasiliensis” must be changed to “S. schenckii”

Supplementary tables 2 and 3 – Tables are incorrectly labelled as in the supplementary files, please correct.

In-text references

Line 69 – Provide reference for soil/ plant association

Line75/83 – Provide reference for differences in virulence

Line 88 – Provide reference for “most closely related species is S. globosa”

Line 93 – Provide reference for “variation of pigmentation and growth rate”

Line 156 – Instead of “(see[10])”, consider “[10]”

Lines 216/217 – Provide references for statistical tests

Lines 225/226 – Cite article that reported MICs

Reference list

Please add relevant issue/ page number information for reference:

1

Please correct title capitalisation in the following references: 6,7,19,20,32,41,44,46,51,52,53,55,56

Please provide abbreviated journal name for the following:

3,49,54

The inclusion of “Epud” dates in some of the reference list is inconsistent. Please remove these from the references.

Reviewer #3: Accept

**********

**Summary and General Comments**

Use this section to provide overall comments, discuss strengths/weaknesses of the study, novelty, significance, general execution and scholarship. You may also include additional comments for the author, including concerns about dual publication, research ethics, or publication ethics. If requesting major revision, please articulate the new experiments that are needed.

Reviewer #1: The manuscript analyzes a relevant subject, the genetic variations of Sporothrix species. In a strict sense, this is not the first approach to address this subject. However, the plus side of this study is the use of whole-genome information to reach the main conclusions. Moreover, the study, unlike others, includes a significant amount of analyzed genomes.

The main shortcomings are:

The lack of an inclusive approach to gather strains from around the world. The sequences are basically from America (South America), and the strains may not have the same genomic features as isolates from Asia or Africa, for example.

Even though the authors focused the analysis on relevant genomic features, the analysis is limited in terms of all the information that may be extracted from a genomic analysis. The gain and loss of putative genes are described and analyzed in a superficial manner. Are there particular loss/gain features in clades? Are there expansions/constrictions in gene families that allow the authors to propose hypotheses on the species-specific traits? A thorough analysis in this an other subjects should be incorporated. The search and distribution of transposable elements is absent, and this has been documented to be a key molecular mechanism in species evolution.

The association between SNP and itraconazole resistance required additional evidence to support the claims included in the manuscript. Experimental evidence showing that a particular change in an SNP renders an itraconazole-sensitive strain into an itraconazole-resistant one should be included as part of the study. How can these SNPs account for the phenotypic change if they are not part of genes? If they belong to a gene, how does the sequence change affect genetic function?

I know that this is a subject of debate between bioinformaticians and researchers based on wet laboratories. Genomic analysis does not allow gene identification. The methodology is robust enough to predict ORFs, which are only a part of a gene. In other words, a gene is not equal to ORF. Moreover, the identification of an ORF does not obligate a sequence to be part of a gene; please keep in mind the presence of pseudogenes. For the sake of research integrity, the auhtors should refer to sequences as predicted ORFS, nor genes. In a similar sense, locus/loci are positions for genes, not for ORFs; the authors should refrain from using these terms.

In the same sense, bioinformatics approach predict functions of putative genes, but whehter there is indeed a transcriptional process on the sequence and whether the RNA undergoes translation are facts not possible to be predicted from the genomic analysis. So, it is wrong to talk about genes encoding X or Y proteins with particular functions. The authors should change the tone to putative ORFs encoding putative proteins with X or Y function.

I did not find the codes of the sequences deposited in bioinformatic repositories.

Reviewer #2: The study "Genome variation of Sporothrix schenckii and Sporothrix brasiliensis" (PNTD-D-26-00623), provides novel insights into the genomic variation of two major Sporothrix pathogens, S. brasiliensis and S. schenckii, highlighting differences in these species that have important epidemiological and evolutionary implications. It is an original and relevant work, that I believe will interest readers of PLOS Neglected Tropical Diseases. However, the suggested revisions, including minor clarification of methodology and limitations, as well as improvements to grammar and referencing, would help strengthen the manuscript further. With these minor revisions, I believe this study will make a valuable contribution to sporotrichosis research.

In terms of data accessibility, accession numbers have been included for all the previously sequenced isolates. However, accession numbers for the sequence data generated in this paper should be updated as they are made available.

Reviewer #3: The manuscript is generally well written and well organized. However, it requires some careful revisions in the Materials and Methods section (see my specific comments).

**********

PLOS authors have the option to publish the peer review history of their article (what does this mean?). If published, this will include your full peer review and any attached files.

If you choose “no”, your identity will remain anonymous but your review may still be made public.

**Do you want your identity to be public for this peer review?**  For information about this choice, including consent withdrawal, please see our Privacy Policy.

Reviewer #1: No

Reviewer #2: **Yes:**  Taygen Fuchs

Reviewer #3: No

[NOTE: If reviewer comments were submitted as an attachment file, they will be attached to this email and accessible via the submission site. Please log into your account, locate the manuscript record, and check for the action link "View Attachments". If this link does not appear, there are no attachment files.]

**Figure resubmission:**

**Reproducibility:**

To enhance the reproducibility of your results, we recommend that authors of applicable studies deposit laboratory protocols in protocols.io, where a protocol can be assigned its own identifier (DOI) such that it can be cited independently in the future. Additionally, PLOS ONE offers an option to publish peer-reviewed clinical study protocols. Read more information on sharing protocols at https://plos.org/protocols?utm_medium=editorial-email&utm_source=authorletters&utm_campaign=protocols

# Author comment: Author response to Decision Letter 1

**DOI:** 10.1371/journal.pntd.0014203.r002

15 Jun 2026

Attachment Submitted filename: Response to Reviewersv3docx.docx

# Peer review: Decision Letter 1

**DOI:** 10.1371/journal.pntd.0014203.r003

**Authors:** Marcio Rodrigues

2 Jul 2026

PNTD-D-26-00623R1

Genome variation of Sporothrix schenckii and Sporothrix brasiliensis

PLOS Neglected Tropical Diseases

Dear Dr. Teixeira,

Thank you for submitting your manuscript to PLOS Neglected Tropical Diseases. At the editorial level, a decision regarding this manuscript proved to be challenging. Two reviewers recommended acceptance, while one reviewer opted to reject it. However, this rejection underscores positive aspects of the manuscript, so we are using the editorial prerogative to invite the authors to revise it. We acknowledge that we concur with the comments made by reviewer 1.

Please submit your revised manuscript within by Aug 31 2026 11:59PM. If you will need more time than this to complete your revisions, please reply to this message or contact the journal office at plosntds@plos.org. When you're ready to submit your revision, log on to https://www.editorialmanager.com/pntd/ and select the 'Submissions Needing Revision' folder to locate your manuscript file.

Please include the following items when submitting your revised manuscript:

* A letter that responds to each point raised by the editor and reviewer(s). You should upload this letter as a separate file labeled 'Response to Reviewers'. This file does not need to include responses to any formatting updates and technical items listed in the 'Journal Requirements' section below.

* A marked-up copy of your manuscript that highlights changes made to the original version. You should upload this as a separate file labeled 'Revised Manuscript with Track Changes'.

* An unmarked version of your revised paper without tracked changes. You should upload this as a separate file labeled 'Manuscript'.

If you would like to make changes to your financial disclosure, competing interests statement, or data availability statement, please make these updates within the submission form at the time of resubmission. Guidelines for resubmitting your figure files are available below the reviewer comments at the end of this letter.

As the corresponding author, your ORCID iD is verified in the submission system and will appear in the published article. PLOS supports the use of ORCID, and we encourage all coauthors to register for an ORCID iD and use it as well. Please encourage your coauthors to verify their ORCID iD within the submission system before final acceptance, as unverified ORCID iDs will not appear in the published article. Only the individual author can complete the verification step; PLOS staff cannot verify ORCID iDs on behalf of authors.

We look forward to receiving your revised manuscript.

Kind regards,

Marcio Rodrigues

Section Editor

PLOS Neglected Tropical Diseases

Shaden Kamhawi

co-Editor-in-Chief

PLOS Neglected Tropical Diseases

orcid.org/0000-0003-4304-636XX

Paul Brindley

co-Editor-in-Chief

PLOS Neglected Tropical Diseases

orcid.org/0000-0003-1765-0002

**Journal Requirements:**

If the reviewer comments include a recommendation to cite specific previously published works, please review and evaluate these publications to determine whether they are relevant and should be cited. There is no requirement to cite these works unless the editor has indicated otherwise.

1) Please amend your detailed Financial Disclosure statement. This is published with the article. It must therefore be completed in full sentences and contain the exact wording you wish to be published.

i) State what role the funders took in the study. If the funders had no role in your study, please state: "The funders had no role in study design, data collection and analysis, decision to publish, or preparation of the manuscript."

ii) If any authors received a salary from any of your funders, please state which authors and which funders..

If you did not receive any funding for this study, please simply state: u201cThe authors received no specific funding for this work.u201d.

**Reviewers' Comments:**

Reviewer's Responses to Questions

**Key Review Criteria Required for Acceptance?**

As you describe the new analyses required for acceptance, please consider the following:

**Methods**

-Are the objectives of the study clearly articulated with a clear testable hypothesis stated?

-Is the study design appropriate to address the stated objectives?

-Is the population clearly described and appropriate for the hypothesis being tested?

-Is the sample size sufficient to ensure adequate power to address the hypothesis being tested?

-Were correct statistical analysis used to support conclusions?

-Are there concerns about ethical or regulatory requirements being met?

Reviewer #1: See below

Reviewer #2: (No Response)

Reviewer #3: The authors have addressed all the technical issues raised during the first round of peer review.

**********

**Results**

-Does the analysis presented match the analysis plan?

-Are the results clearly and completely presented?

-Are the figures (Tables, Images) of sufficient quality for clarity?

Reviewer #1: See below

Reviewer #2: (No Response)

Reviewer #3: The authors have addressed all the issues raised during the first round of peer review.

**********

**Conclusions**

-Are the conclusions supported by the data presented?

-Are the limitations of analysis clearly described?

-Do the authors discuss how these data can be helpful to advance our understanding of the topic under study?

-Is public health relevance addressed?

Reviewer #1: See below

Reviewer #2: (No Response)

Reviewer #3: Overall, this section is clearly presented and the conclusions are adequately supported by the data.

**********

**Editorial and Data Presentation Modifications?**

Use this section for editorial suggestions as well as relatively minor modifications of existing data that would enhance clarity. If the only modifications needed are minor and/or editorial, you may wish to recommend “Minor Revision” or “Accept”.

Reviewer #1: (No Response)

Reviewer #2: (No Response)

Reviewer #3: Accept

**********

**Summary and General Comments**

Use this section to provide overall comments, discuss strengths/weaknesses of the study, novelty, significance, general execution and scholarship. You may also include additional comments for the author, including concerns about dual publication, research ethics, or publication ethics. If requesting major revision, please articulate the new experiments that are needed.

Reviewer #1: I thank the authors for the improvements to the manuscript and for their comments on my observations. As the authors mentioned in the rebuttal letter, there is a lot to do to refine the analysis, and currently, they are sitting on a bunch of interesting data that are waiting for a thorough and robust analysis. As I mentioned in my first assessment, the analysis is superficial and needs more work. The weakest part of the study is related to the putative SNPs linked to antifungal resistance; without additional experimental validation, the authors do not have sufficient evidence to sustain the drawn conclusions. My recommendation is to halt the publication of the study as it is, to invest time to complement it, and then to seek the publication. Such a version would be a strong contribution to the field.

Reviewer #2: The article "Genome variation of Sporothrix schenckii and Sporothrix brasiliensis" is an interesting and informative study that expands current knowledge on two major Sporothrix pathogens. Using genomic approaches the authors explored the genetic diversity of the two Sporothrix pathogens, and related these insights back to epidemiological data, providing valuable insights into their population structure, reproduction and dissemination. This work is timely considering the current state of sporotrichosis and the growing relevance of feline driven outbreaks in both pathogens. Furthermore, the genomic data generated in this study provide valuable resources for future genetic diversity and comparative genomic studies. The authors adequately considered all the suggestions from the last round of review and I believe the article is suitable for publication in the current form.

Reviewer #3: (No Response)

**********

PLOS authors have the option to publish the peer review history of their article (what does this mean?). If published, this will include your full peer review and any attached files.

If you choose “no”, your identity will remain anonymous but your review may still be made public.

**Do you want your identity to be public for this peer review?**  For information about this choice, including consent withdrawal, please see our Privacy Policy.

Reviewer #1: No

Reviewer #2: No

Reviewer #3: No

[NOTE: If reviewer comments were submitted as an attachment file, they will be attached to this email and accessible via the submission site. Please log into your account, locate the manuscript record, and check for the action link "View Attachments". If this link does not appear, there are no attachment files.]

**Figure resubmission:**

**Reproducibility:**

To enhance the reproducibility of your results, we recommend that authors of applicable studies deposit laboratory protocols in protocols.io, where a protocol can be assigned its own identifier (DOI) such that it can be cited independently in the future. Additionally, PLOS ONE offers an option to publish peer-reviewed clinical study protocols. Read more information on sharing protocols at https://plos.org/protocols?utm_medium=editorial-email&utm_source=authorletters&utm_campaign=protocols

# Author comment: Author response to Decision Letter 2

**DOI:** 10.1371/journal.pntd.0014203.r004

28 Jul 2026

Attachment Submitted filename: ResponsetoReviewersR2.docx

# Editor report: Decision Letter 2

**DOI:** 10.1371/journal.pntd.0014203.r005

**Authors:** Marcio Rodrigues

2 Aug 2026

Dear Mr. Teixeira,

We are pleased to inform you that your manuscript 'Genome variation of Sporothrix schenckii and Sporothrix brasiliensis' has been provisionally accepted for publication in PLOS Neglected Tropical Diseases.

Before your manuscript can be formally accepted you will need to complete some formatting changes, which you will receive in a follow up email. A member of our team will be in touch with a set of requests.

Please note that your manuscript will not be scheduled for publication until you have made the required changes, so a swift response is appreciated.

IMPORTANT: The editorial review process is now complete. PLOS will only permit corrections to spelling, formatting or significant scientific errors from this point onwards. Requests for major changes, or any which affect the scientific understanding of your work, will cause delays to the publication date of your manuscript.

Should you, your institution's press office or the journal office choose to press release your paper, you will automatically be opted out of early publication. We ask that you notify us now if you or your institution is planning to press release the article. All press must be co-ordinated with PLOS.

Thank you again for supporting Open Access publishing; we are looking forward to publishing your work in PLOS Neglected Tropical Diseases.

Best regards,

Marcio L Rodrigues

Section Editor

PLOS Neglected Tropical Diseases

Marcio Rodrigues

Section Editor

PLOS Neglected Tropical Diseases

Shaden Kamhawi

co-Editor-in-Chief

PLOS Neglected Tropical Diseases

orcid.org/0000-0003-4304-636XX

Paul Brindley

co-Editor-in-Chief

PLOS Neglected Tropical Diseases

orcid.org/0000-0003-1765-0002

***********************************************************

# Editor report: Acceptance letter

**DOI:** 10.1371/journal.pntd.0014203.r006

**Authors:** Marcio Rodrigues

Dear Mr. Teixeira,

We are delighted to inform you that your manuscript, "Genome variation of Sporothrix schenckii and Sporothrix brasiliensis," has been formally accepted for publication in PLOS Neglected Tropical Diseases.

We have now passed your article onto the PLOS Production Department who will complete the rest of the publication process. All authors will receive a confirmation email upon publication.

The corresponding author will soon be receiving a typeset proof for review, to ensure errors have not been introduced during production. Please review the PDF proof of your manuscript carefully, as this is the last chance to correct any scientific or type-setting errors. Please note that major changes, or those which affect the scientific understanding of the work, will likely cause delays to the publication date of your manuscript. Note: Proofs for Front Matter articles (Editorial, Viewpoint, Symposium, Review, etc...) are generated on a different schedule and may not be made available as quickly.

Soon after your final files are uploaded, the early version of your manuscript will be published online unless you opted out of this process. The date of the early version will be your article's publication date. The final article will be published to the same URL, and all versions of the paper will be accessible to readers.

For Research Articles, you will receive an invoice from PLOS for your publication fee after your manuscript has reached the completed accept phase. If you receive an email requesting payment before acceptance or for any other service, this may be a phishing scheme. Learn how to identify phishing emails and protect your accounts at https://explore.plos.org/phishing.

Thank you again for supporting open-access publishing; we are looking forward to publishing your work in PLOS Neglected Tropical Diseases.

Best regards,

Shaden Kamhawi

co-Editor-in-Chief

PLOS Neglected Tropical Diseases

Paul Brindley

co-Editor-in-Chief

PLOS Neglected Tropical Diseases
